# Cellphone enabled point-of-care assessment of breast tumor cytology and molecular HER2 expression from fine-needle aspirates

**DOI:** 10.1038/s41523-021-00290-0

**Published:** 2021-07-02

**Authors:** Daniel Y. Joh, Jacob T. Heggestad, Shengwei Zhang, Gray R. Anderson, Jayanta Bhattacharyya, Suzanne E. Wardell, Simone A. Wall, Amy B. Cheng, Faris Albarghouthi, Jason Liu, Sachi Oshima, Angus M. Hucknall, Terry Hyslop, Allison H. S. Hall, Kris C. Wood, E. Shelley Hwang, Kyle C. Strickland, Qingshan Wei, Ashutosh Chilkoti

**Affiliations:** 1 Department of Biomedical Engineering, Pratt School of Engineering, Duke University, Durham, NC USA; 2 Division of Plastic, Maxillofacial, and Oral Surgery, Department of Surgery, Duke University Medical Center, Durham, NC USA; 3 Department of Chemical and Biomolecular Engineering, North Carolina State University, Raleigh, NC USA; 4 Department of Pharmacology and Cancer Biology, Duke University School of Medicine, Durham, NC USA; 5 Center for Biomedical Engineering, Indian Institute of Technology, Hauz Khas, New Delhi, 110016 India; 6 Division of Surgical Oncology, Department of Surgery, Duke University Medical Center, Durham, NC USA; 7 Department of Biostatistics & Bioinformatics, Duke University Medical Center, Durham, NC USA; 8 Department of Pathology, Duke University Medical Center, Durham, NC USA

**Keywords:** Diagnostic markers, Pathology, Translational research

## Abstract

Management of breast cancer in limited-resource settings is hindered by a lack of low-cost, logistically sustainable approaches toward molecular and cellular diagnostic pathology services that are needed to guide therapy. To address these limitations, we have developed a multimodal cellphone-based platform—the EpiView-D4—that can evaluate both cellular morphology and molecular expression of clinically relevant biomarkers directly from fine-needle aspiration (FNA) of breast tissue specimens within 1 h. The EpiView-D4 is comprised of two components: (1) an immunodiagnostic chip built upon a “non-fouling” polymer brush-coating (the “D4”) which quantifies expression of protein biomarkers directly from crude cell lysates, and (2) a custom cellphone-based optical microscope (“EpiView”) designed for imaging cytology preparations and D4 assay readout. As a proof-of-concept, we used the EpiView-D4 for assessment of human epidermal growth factor receptor-2 (HER2) expression and validated the performance using cancer cell lines, animal models, and human tissue specimens. We found that FNA cytology specimens (prepared in less than 5 min with rapid staining kits) imaged by the EpiView-D4 were adequate for assessment of lesional cellularity and tumor content. We also found our device could reliably distinguish between HER2 expression levels across multiple different cell lines and animal xenografts. In a pilot study with human tissue (*n* = 19), we were able to accurately categorize HER2-negative and HER2-positve tumors from FNA specimens. Taken together, the EpiView-D4 offers a promising alternative to invasive—and often unavailable—pathology services and may enable the democratization of effective breast cancer management in limited-resource settings.

## Introduction

Breast cancer is a major global health concern. It is the most common cancer among women and the leading cause of cancer death for women worldwide^1^. Limited-resource settings now account for about half of the cases and the majority of deaths from breast cancer, and the mortality gap between developed and underdeveloped countries continues to widen^1,2^. One major reason for poor outcomes in limited resource settings is related to the higher incidence of late-stage diagnosis resulting from the lack of healthcare infrastructure to support diagnostic pathology services^3–5^.

Diagnostic pathology is a cornerstone of breast cancer management, and the quality of pathology services directly correlates with the quality of care and patient outcomes^4^. In abundant-resource settings, breast pathology is performed by trained individuals in well equipped, centralized facilities. Surgically excised specimens or core-needle biopsies (CNB) are processed using standard methods that allow both histopathology and molecular pathology on stained tissue sections. Initial morphologic evaluation of histopathology allows for diagnosis of malignancy and pathologic staging of surgical specimens^6^. Molecular pathology evaluation, typically by immunohistochemistry (IHC), identifies expression levels of several key biomarkers: estrogen receptor (ER), progesterone receptor (PR), human epidermal growth factor receptor-2 (HER2), and the proliferation marker Ki67; these markers are critical for disease subtyping, prognosis, and for selecting therapies to which tumor subtypes are most likely to respond^7–9^. This “personalized” treatment approach based on biomarker expression has driven a dramatic improvement in survival in abundant-resource settings over the past several decades^8–19^. Unfortunately, this level of diagnostic information is often unattainable in limited resource settings, resulting in treatment decisions based on little to no pathology guidance, or prolonged turnaround times for pathology results that risks disease progression or no follow up^4,20–23^. Although several mainstay therapies including anti-hormone treatment and targeted drugs such as trastuzumab are now on the WHO Essential Medications Lists^24–26^, treatment by these drugs is of limited use if it is not guided by appropriate diagnostic pathology. Hence, without the ability to accurately identify and subtype breast cancers, the mortality gap between developed and underdeveloped countries is expected to remain wide^27^.

Currently, Resource Stratified Guidelines by the Breast Health Global Initiative (BHGI) emphasizes the use of fine-needle aspirate (FNA) biopsies —as opposed to CNB— as the preferred approach for initial pathological investigation in limited-resource settings^3,4,6,28^. While FNA has shortcomings compared to CNB, (e.g., lack of information about tissue architecture), it is advantageous for limited resource settings because it: (1) permits direct assessment of malignancy without cell-block processing methods, (2) is less invasive than CNB, and (3) can be performed at the bedside or outpatient setting by technicians using readily available equipment such as syringes and rapid cytology stains^29,30^. However, the effectiveness of FNA-based pathology in limited-resource settings is limited by two major issues. First, cytopathology evaluation still depends on the availability of on-site pathologists and ultimately the use of a microscope to interpret cytology^6^, which poses logistical challenges. Second, FNA is limited to morphologic evaluation without providing key information on molecular biomarker expression that is necessary for treatment selection^7,31^. While biomarker assessment of FNA preparations by “immunocytochemistry (ICC)” is possible in some laboratories^32–34^, infrastructure constraints and difficulty with standardization usually precludes this option in limited resource settings.

An exciting advancement to this end is the recently described CytoPAN platform, a portable fluorescence cytometer that enables molecular assessment of ER/PR expression in FNA specimens within 1 h at low-cost^35^. This method is a step forward toward expanding access to breast pathology, but its limitations include the need to refrigerate assay kits, the need for multiple immunostaining steps (blocking, centrifugation, washing, staining, etc.), and the absence of morphologic (cytology) assessment of tumor cells. Although the CytoPAN is a welcome advance, it also highlights the continuing and urgent need for new methods for morphologic and biomarker assessment of tumor specimens in limited-resource settings that are technologically even simpler, require minimal infrastructure, and minimal user training to operate.

**Fig. 1:**
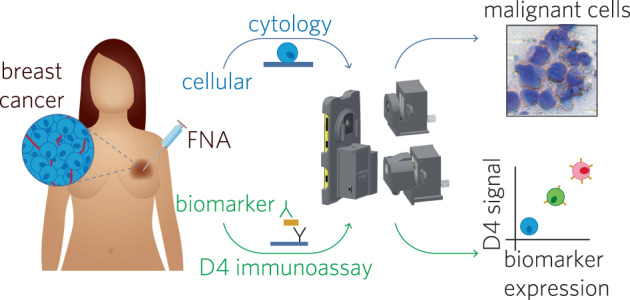
Schematic representation of the EpiView-D4 mobile pathology platform for breast cancer. The device has a smartphone base which uses detachable modules for brightfield (top) and fluorescence (bottom) imaging using the cell phone camera. Tumor is first sampled by FNA, and then aspirates are processed for cytology and biomarker evaluation. For cytology, aspirates are smeared on a glass slide and processed with rapid (DiffQuik^TM^) staining kits and then imaged with the brightfield imaging attachment (top). For biomarker evaluation, aspirates are mixed with lysis buffer and then applied to a D4 immunoassay chip, which quantify an analyte of interest (in this case, HER2). D4 chips are read using the fluorescence imaging attachment. The fluorescence intensity of the cAb spots on the D4 chip correlates with the biomarker expression level.

Herein, we describe a multimodal mobile pathology platform —the “EpiView-D4”— that meets these criteria (Fig. 1). It assesses both biomarker expression and cytology from FNA specimens on a user friendly and portable platform that is much cheaper than standard laboratory methods. For this paper, we chose to measure HER2 expression because of its clinical significance, as one-fourth of all breast cancers overexpress HER2, and anti-HER2 targeted therapies substantially lower the risk of recurrence and death for HER2-positive disease^11–17^. The EpiView-D4 consists of two components: (1) a self-contained immunodiagnostic chip (the “D4”) for quantification of HER2, and (2) a custom cellphone-based optical microscope (the “EpiView”) that performs both brightfield imaging of basic cytology preparations and fluorescent readout of the D4 chip.

The paper is organized as follows: first, we assess the analytical performance of the D4 assay in simulated samples spiked with HER2 protein, and then quantify the expression level of HER2 in lysed tumor cells from cell culture. Next, we describe the design, and demonstrate the performance of the EpiView mobile microscopy platform for both brightfield imaging for cytology and fluorescence readout of the D4 chip. Combining these two elements as the EpiView-D4, we then characterize the cytology and HER2 expression of FNA specimens from orthotopically engrafted solid tumor models of human breast cancer in mice. Finally, we demonstrate the translational relevance of the EpiView-D4 for breast cancer diagnosis and biomarker profiling by analysis of clinical breast cancer specimens from human patients at Duke University Medical Center (DUMC). Our results from these studies suggest that the EpiView-D4 has the potential to make breast pathology services broadly accessible in limited-resource settings.

## Results

### The “D4”— a polymer brush-based immunodiagnostic chip for credentialing HER2 biomarker expression by breast cancer cells

**Fig. 2:**
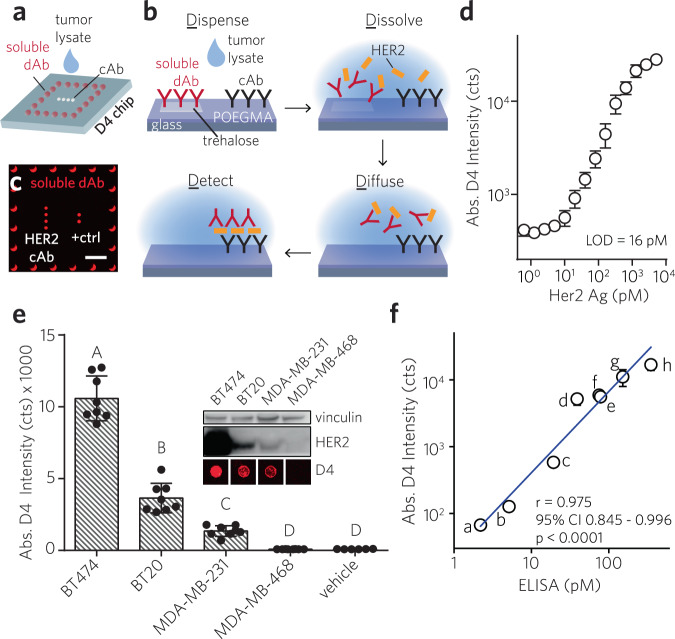
D4 sandwich immunoassay for quantification of HER2 expression level and its in vitro assessment. **a**–**c**, Schematic and operation of D4 immunoassay. **a** Spots of immobile cAb and an excess of “soluble” fluorescently labeled dAb are printed directly onto POEGMA-coated glass. **b** Dispensing sample fluid onto chip surface leads to dissolution of soluble dAb spots, followed by diffusion-driven mixing and antibody “sandwich” formation if HER2 is present. HER2 binding is detected by fluorescence imaging. **c** Representative fluorescence image of D4 assay after exposure to HER2-spiked RIPA buffer. Scale bar, 1 mm. **d** Representative dose-response curve generated from D4 chips for RIPA buffer spiked with recombinant HER2. Error bars: mean ± s.d. of duplicate assays. **e**, Assessment of cultured breast cancer cell lines (BT474, BT20, MDA-MB-231, MDA-MB-468) by D4 assay and comparison with western blotting. Inset: Western blot against HER2 for each cell line, with representative D4 cAb spots underneath. Main: D4 signal intensity (mean ± 95% CI) of ≥6 replicates. Significant difference by one-way ANOVA (F(4, 31) = 179.4, *p* < 0.0001). Bars with different letters indicate different groups (Tukey post hoc test, *p* ≤ 0.05). **f** Concordance analysis of 8 different patient-derived tumor cell lines in culture for HER2 expression by D4 assay vs. ELISA performed by clinical lab; specimens indexed “a” through “h”. D4 results are mean ± s.d. of duplicate assays. Pearson’s *r* = 0.975 (*p* < 0.0001, 95% CI: 0.845–0.996).

Our strategy to quantify the expression of HER2 builds upon the “D4” assay platform we recently developed and reported elsewhere^36^. In brief, the D4 is a self-contained sandwich immunoassay platform fabricated by printing an antibody (Ab) pair onto a glass substrate coated with a “nonfouling” (protein- and cell- resistant) polymer film (Fig. 2a, b). This polymer film is ~50 nm thick and is composed of a poly(oligoethylene glycol methyl ether methacrylate) (POEGMA) polymer brush grown from the surface by surface-initiated atom transfer radical polymerization (SI-ATRP)^37–43^. All antibody reagents are directly inkjet printed on the POEGMA coating by a non-contact piezo printer. Unlabeled capture Abs (cAbs) are printed directly onto the POEGMA brush as an array of microspots, while fluorescently tagged detection Abs (dAbs) are printed with an excess of added excipient —trehalose— as a corral of microspots that surround the column of cAb spots. Trehalose is added to ensure dissolution of the dAb upon contact with an aqueous fluid such as tumor lysate or blood. Testing a sample tumor cell lysate for HER2 entails applying tumor lysate directly to the assay surface (Fig. 2b). This hydrates the surface, which dissolves the dAb from the printed spots, liberating it into solution and also enables diffusion-driven mixing of reagents, leading to the formation of an Ab “sandwich” at the location of the cAb spots. The assay is read by imaging the fluorescence from the cAb microspots with a fluorescence detector, which converts the fluorescence intensity to HER2 concentration (Fig. 2c). Given the similar complexity of blood and tumor lysate as sample matrices, and our success in quantifying soluble protein analytes from blood with the D4 assay, we hypothesized that a D4 assay for HER2 should be able to quantify the receptor expression level from crude lysate generated from breast tumor cells without any further processing.

We first carried out a HER2 sandwich immunoassay on the D4 chip using various amounts of recombinant HER2 spiked into radioimmunoprecipitation assay (RIPA) buffer, and quantified the fluorescence intensity of the microspots on the D4 chip with a tabletop fluorescence scanner. We chose RIPA buffer as the vehicle, as it is a widely used agent to lyse mammalian cells and extract and solubilize membrane-bound proteins, like HER2. Fig. 2d shows a representative dose-response curve for HER2, with a limit of detection (LOD) of ~16 pM and a dynamic range spanning over 3 orders of magnitude.

Next, we tested the D4 HER2 assay against four established human breast cancer cell lines: BT474, BT20, MDA-MB-231, and MDA-MB-468 (Fig. 2e). We chose these cell lines because the first three have varying levels of HER2 expression, while MDA-MB-468 does not express HER2, and hence serves as a negative control. The relative HER2 expression levels in these cell lines were first confirmed by western blotting (inset, Fig. 2e), and established the rank order of HER2 expression as BT474 > BT20 > MDA-MB-231 > MDA-MB-468, consistent with prior observations^44,45^. Each cell line was harvested by trypsinization and incubated in RIPA buffer (20,000 cells per μL), and the crude lysate were directly applied to the D4 chip and read by a Genepix fluorescence scanner. We chose to use a tabletop scanner for fluorescence readout of the HER2 D4 chips in these characterization experiments, as it has a high sensitivity and dynamic range and allows us to decouple the performance of the D4 assay from the portable EpiView detector. Representative fluorescence images of cAb spots after incubation with cell lysate visually show that their intensities are consistent with the relative HER2 expression levels for each cell line observed by western blotting (inset, Fig. 2e). Quantitation of the fluorescence intensity (bar graph in Fig. 2e) shows that the D4 assay can quantitatively distinguish between the HER2 levels in the different cell lines and the D4 assay readout exhibits the same rank ordering of HER2 expression as observed by western blotting.

In addition to testing commercially available cell lines, we also tested 8 different patient-derived tumor cell lines (PDTCs). Breast cancer specimens surgically obtained from patients treated at DUMC were propagated in vitro as conditionally reprogrammed cells in the presence of a Rho kinase inhibitor and feeder cells^46^. Lysates of cultured PDTCs were generated using similar procedures as above, and were then quantitatively assessed by the D4 (samples labeled “a–h”), and were evaluated in parallel by a HER2 ELISA run separately by staff at a centralized laboratory (Stedman Immunoassay Laboratory at DUMC) (Fig. 2f). The D4 readout has a strong positive correlation with ELISA across all 8 pairs of measurements, with a Pearson’s *r* of 0.975 (*p* < 0.0001, 95% CI: 0.845–0.996).

We next confirmed the specificity of the HER2 D4 assay by two independent methods. First, we examined the “molecular specificity” of the D4 signal by using genome editing to modulate the expression levels of HER2 in a cell line (Supplementary Fig. 1a). Using CRISPR-Cas9 technology, we generated stable pools of BT474 cells with HER2 knock-down using two independent sgRNAs (sgHER2 #1, #2). In general, we see that both BT474 knock-down cells have decreased levels of HER2 protein when compared to cells that were transduced with a control sgRNA (ctrl sgRNA). As shown by the corresponding representative fluorescence images of HER2 cAb microspots, the D4 assay confirms the expression levels seen via western blotting, namely, that HER2 expression is nearly absent in the sgHER2 #1 pool, and decreased in the sgHER2 #2 pool, both relative to the control cells. Quantitative values of D4 fluorescence intensities for each condition are displayed in Supplementary Fig. 1b. Second, we stably over-expressed HER2 in MDA-MB-468—a cell line that lacks expression of HER2—under a constitutive promoter. The D4 assay shows robust overexpression of HER2 in the HER2 transfected cells when compared to a negative control—cells that overexpress luciferase. The D4 assay results are consistent with Western blotting, and clearly show the molecular specificity of the HER2 D4 assay.

We also examined the influence of “matrix effects” in cell specimens due to the presence of non-breast cancer cells in the tumor microenvironment, as the FNA from which the tumor lysate is generated contains a mixture of cancerous and healthy cells. To do so, we compared the fluorescence response of the D4 assay in the presence and absence of NIH 3T3 fibroblasts in the tumor lysate. The dose-response curve for a D4 assay carried out on lysate from HER2-positive BT474 cells in RIPA is shown in Supplementary Fig. 1c. The LOD of the D4 assay was determined from the dose-response curve to be 12 cells/µL. Next, we mixed 3T3 fibroblasts with the BT474 cancer cells and carried out a D4 assay. Supplementary Fig. 1d shows the dose-response curve for BT474 in in the presence of 3T3 fibroblasts, and shows that the D4 assay has a LOD of 14 cells/µL, similar to that measured previously for a lysate of BT474 cells only. Together, these results indicate that the analytical sensitivity of the D4 assay is not affected by healthy cells in the lysate. The high sensitivity and specificity for HER2 in the D4 assay can be attributed to the specificity of the Ab’s used for the assay and the nonfouling properties of the POEGMA coating, which prevents adventitious surface biomolecular interactions that may interfere with the assay^36,37,39^.

Having developed and characterized a functional D4 assay capable of detecting HER2 in crude cellular lysates using a tabletop fluorescence scanner for readout, we next describe the development of a dual-function mobile platform that allows brightfield imaging of cytology specimens and fluorescence readout of the D4 assay.

### The “EpiView”— a modular, dual-function smartphone microcopy platform for brightfield imaging and fluorescence quantification

**Fig. 3:**
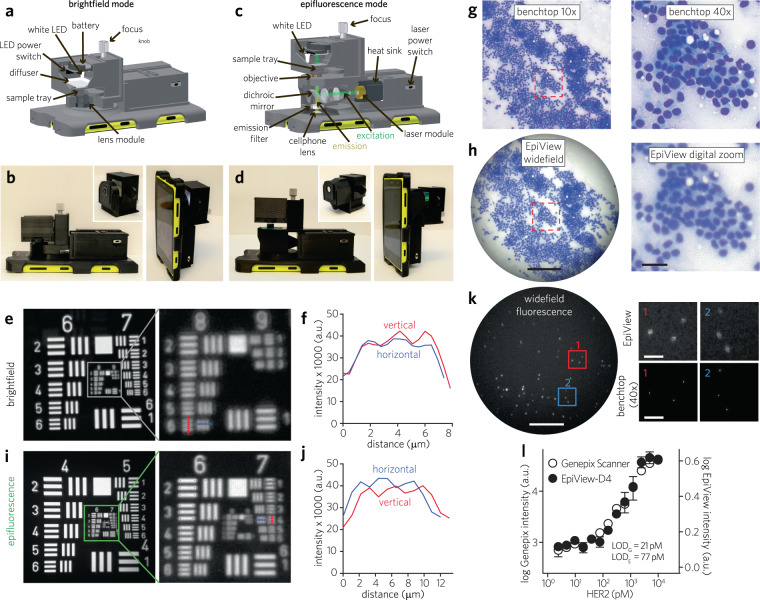
EpiView mobile microscope for brightfield and fluorescence imaging. **a–d** Optomechanical design of EpiView. Labeled 3D schematic and photographs of the EpiView scope with brightfield (**a**, **b**) and epifluorescence (**c**, **d**) assemblies. **e**, **f** Raw resolution of brightfield imaging from unprocessed images of USAF 1951 test target. Brightfield images of test target (green channel extracted) are shown in **e**, with red and blue dashed lines in image corresponding to vertical (red) and horizontal (blue) intensity profiles of test target features in **f**. Comparison of brightfield imaging of FNA cytology from human breast cancer specimen using standard benchtop microscopy (**g**) and EpiView device (**h**). Left panels: standard microscope image obtained by a 10× objective lens (top) and native view on EpiView (bottom). Scale bar, 0.2 mm. Right panels: standard microscope image obtained by a 40× objective (top), showing the same ROI outlined with red dashed line in the 10× image, and similar view obtained by digital zoom on EpiView (bottom). Scale bar, 30 µm. **i**, **j** Resolution testing with USAF 1951 in a manner similar to **e** and **f** but in epifluorescence mode based on extracting green channel image. **k** Left panel: Widefield view of 200 nm fluorescence beads using EpiView in epifluorescence mode. Scale bar, 0.1 mm. Right panel: Comparison of EpiView versus and benchtop microscope (40× objective, NA = 0.6) of the 200 nm beads outlined by the red and blue ROIs in the widefield image. Images were extracted from green channel. Scale bars, 40 µm. **l** Dose-response curve of HER2 with D4-EpiView and a conventional glass slide scanner. Limit of detection for this experiment using conventional glass scanner (LOD_G_) is 21 pM, while that of the D4-EpiView (LOD_E_) is 77 pM. Data represent log signal intensity ± s.d for duplicate assays for both EpiView-D4 and the slide scanner.

To complement the D4 chip, we concurrently developed the EpiView — a new smartphone-based imaging platform. We designed the EpiView as a mobile microscope that performs two key functions on a single platform: (1) visualizes FNA cytology specimens, and (2) quantifies the fluorescence output of D4 assay chips for HER2 evaluation. The major design challenge we had to overcome was to develop a high-quality handheld imaging device that could rapidly switch between brightfield and fluorescence modes without extensive re-assembly or recalibration. To accomplish this task, we developed a modular device, where switchable imaging units—one for brightfield imaging and another for fluorescence imaging—attach to a common base unit that is mounted on a smartphone (Fig. 3a–d). The base unit contains a laser diode and batteries for the fluorescence imaging modality. The brightfield imaging attachment (Fig. 3a, b) is based on a transmission illumination design that uses a white LED as the illumination source. The module also integrates a small translational stage connected to the sample tray, an optical diffuser, and an external lens module to provide the necessary optical magnification. The design of the fluorescence imaging attachment utilizes an epifluorescence optical configuration (Fig. 3c, d). In this design, a dichroic mirror placed at 45° to the incident light is used as a beam splitter to deflect excitation light from a compact laser diode and to allow emitted photons from the sample to pass through to the smartphone camera (Fig. 3c). In addition to the focusing stage and emission filter, the imaging module also includes a beam expansion unit consisting of two singlet lenses to expand the diameter of laser beam by around 2.6× to fill the back aperture of the objective lens module. A white LED is also incorporated for illumination to facilitate sample searching and initial focusing.

We first tested the performance of the brightfield imaging attachment. Using the setup shown in Fig. 3a, b, we achieved a raw spatial resolution of 1.23 μm while simultaneously maintaining a large field of view (FOV) of ∼0.8 mm^2^ in brightfield mode (Fig. 3e, f). To further improve resolution, the point spread function (PSF) of the imaging system was measured and the raw brightfield images were deconvoluted in RGB color space, which improved the spatial resolution down to 1.1 μm (see Supplementary Fig. 2 and Methods). In Fig. 3g, h, we show representative brightfield images of FNA cytology from a breast tumor specimen excised from a human subject, taken using a standard brightfield microscope and the EpiView in both low- and high-power fields (LPF and HPF, respectively). Our device easily permits scanning the specimen at LPF with image quality comparable to a standard optical microscope (left panel, Fig. 3g, h). Notably, HPF images on the EpiView are obtained by digitally zooming on a widefield image, rather than needing to switch objective lenses as is required on a microscope. Although the benchtop microscope exhibited better resolution and contrast for HPF images, the digitally magnified images obtained by our device were deemed sufficient to confirm cellularity of the sample based on review by board-certified pathologists (right panel, Fig. 3g, h) with the added advantage of recording all data in widefield mode without needing to switch lenses for increased magnification.

We next evaluated the performance of the EpiView fluorescence imaging attachment. The fluorescence imaging module provides both a large FOV (∼1.5 mm^2^) and a fine (raw) lateral resolution of 2.6 µm (Fig. 3i, j). To demonstrate the sensitivity of the device in epifluorescence mode, we imaged fluorescent polystyrene beads with sub-micron diameter as shown in Fig. 3k and Supplementary Fig. 3 (bead diameter = 200 nm and 100 nm, respectively). Most of the fluorescent signal captured by the EpiView are of single beads scattered at low density across a glass slide, as confirmed by a standard inverted microscope equipped with a 40× (NA = 0.6) or 100× (NA = 1.4) objective lens for 200 nm and 100 nm beads (respectively). In our previously published work, we introduced a smartphone microscope based on oblique, focused illumination that was capable of imaging beads with diameters down to 100 nm^47^. In epifluorescence mode, the EpiView matches the imaging sensitivity of our previous embodiment, with the additional advantage of more uniform illumination across a wide FOV (as opposed to focused illumination), translating to a more robust platform.

Having developed a robust epifluorescence imaging module, we confirmed the ability of the EpiView to quantify the fluorescence signal from D4 assay chips. Fig. 3l shows dose-response curves for a D4 assay for HER2 in RIPA, and quantified with the EpiView and a tabletop scanner. The EpiView has a LOD of 77 pM, while that of the scanner is 21 pM, which translates to 3.6-fold lower sensitivity for the EpiView as compared to the tabletop scanner. To place these results in context, our older smartphone fluorescence microscope had a ~20-fold lower analytical sensitivity than the tabletop scanner^36^.

### The combined “EpiView-D4”— assessment of solid tumors from mice engrafted with human breast cancer lines

**Fig. 4:**
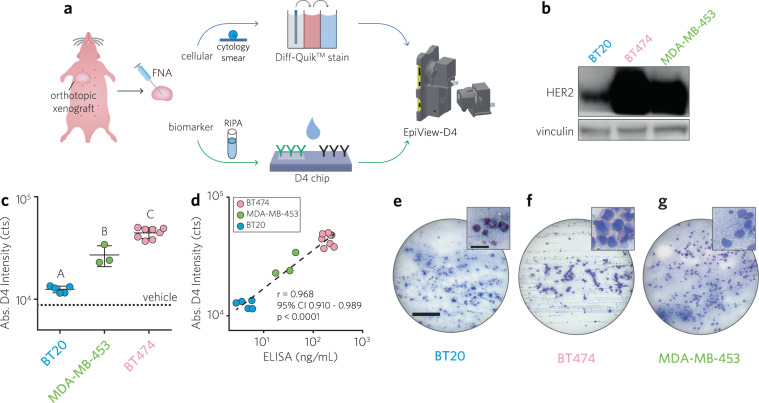
Evaluation of solid tumor xenografts from nude mice orthotopically engrafted with human breast cancer cell lines with EpiView-D4. **a** Schematic of workflow. Tumor aspirates are divided into two aliquots; one aliquot from an aspirate is processed as a cytology specimen on a glass slide using a Diff-Quik^TM^ rapid staining kit and then imaged using the brightfield imaging attachment (top row), The second aliquot is lysed in RIPA buffer and applied to a D4 chip and its HER2 level is quantified using the fluorescence attachment on the EpiView-D4. **b** Representative western blots against HER2 for each human breast cancer cell line used for xenografting (BT20, BT474, and MDA-MB-453). Vinculin used as loading control. **c** Results of HER2 credentialing for 16 different solid tumor specimens from BT20, BT474, and MDA-MB-453 xenografts (*N* = 5, 8, 3, respectively). Each data point represents the D4 fluorescence intensity (average of duplicates) measured by the EpiView-D4 for individual mouse tumors, categorized by xenograft line. Also shown is the mean ± 95% CI fluorescence intensity for each category, which mirrors the western blots shown in panel **b**. There was a statistically significant difference between groups as determined by one-way ANOVA (F (2, 13) = 76.10, *p* < 0.0001). Multiple comparison testing showed significant differences between each group (Tukey post hoc test, *p* ≤ 0.05). **d** D4 fluorescence intensity by EpiView-D4 for each mouse tumor from **f** plotted against corresponding HER2 ELISA. Pearson *r* = 0.968, *p* < 0.0001, 95% CI: 0.910–0.989). **e**–**g** Representative LPF (main panel) and HPF (inset) images of FNA preparations for each xenograft line confirm malignant cytology (see Supplementary Fig. 4 for representative images with standard microscope). Scale bars for LPF and HPF images are 0.2 mm and 30 µm, respectively.

Having characterized the performance of the D4 chip and the dual-function imaging capability of the EpiView, we next sought to test the ability of the combined platform—the EpiView-D4—to assess both cytomorphology of FNA preparations and HER2 expression in animal models of human breast cancer (Fig. 4a). To this end, we tested solid tumor xenografts derived from nude mice that were orthotopically implanted with BT474, MDA-MB-453, or BT20 tumor cells in the axial mammary fat pads (*N* = 8, 3, 5, respectively). As depicted in the western blot in Fig. 4b, the rank ordering of HER2 expression levels for each cell line is BT474 > MDA-MB-453 > BT20, consistent with prior observations^44,45^. For comparison, IHC staining of xenografts against HER2 by standard methods was also performed on representative xenografts and imaged using both a microscope and the EpiView-D4 (Supplementary Fig. 4). When reviewed by a pathologist (KS), IHC of BT474 tumor specimens scored as HER2-positive (3+), while both MDA-MB-453 and BT20 specimens both scored as clinically equivocal (2+) for HER2; we speculate the difficulty in distinguishing HER2 expression in the latter two specimens compared to western blotting relates to the known variability of IHC results that may occur based on factors such as reagents used, epitope accessibility, and staining technique^48–50^.

To assess these solid tumor xenografts on the EpiView-D4, we sampled them by the Zajdela —capillary action— FNA technique using 23-gauge needles^51^. This approach typically yielded ~1 mg of sample per FNA. One aliquot was mixed with RIPA at a density of 1 mg/100 µL and then applied to D4 chips to determine the HER2 expression level. The other aliquot was used to prepare cytology smears on glass slides that were immediately processed with a Diff-Quik^TM^ rapid staining kit (processing time <5 min).

There was a statistically significant difference in the HER2 expression levels as quantified by the EpiView-D4 assay between BT474, MDA-MB-453, and BT20 xenografts as determined by one-way ANOVA (F (2, 13) = 76.10, *p* < 0.0001) and Tukey’s post hoc testing, (*p* ≤ 0.05) (Fig. 4c). These results are qualitatively consistent with the Western blots in Fig. 4b. The tumor lysates were also quantified for their HER2 expression levels by standard ELISA that was run by Stedman Immunoassay Laboratory at DUMC and compared with D4 results. There was a statistically significant linear correlation between the HER2 expression level as measured by the EpiView-D4 and ELISA (Pearson *r* = 0.968, *p* < 0.0001, 95% CI: 0.910–0.989). (Fig. 4d).

Representative LPF and HPF brightfield images of cytopathology taken by the EpiView-D4 for each xenograft are shown in Fig. 4e–g. For comparison, similar images taken with a conventional brightfield microscope are shown in Supplementary Fig. 4. These images were reviewed by a cytopathologist (KS), who confirmed that the images taken by the EpiView-D4 were adequate to clinically evaluate lesional cellularity and tumor content.

### Evaluation of tumor specimens from breast cancer patients at DUMC

Finally, we investigated the use of the EpiView-D4 in breast cancer tissues obtained from human subjects. To do so, we obtained 19 de-identified, fresh-frozen tissue specimens from breast cancer patients treated by the surgical oncology group at DUMC. Of note, these included both non-tumor and tumor specimens with varying HER2 status that were provided in a blinded fashion (Supplementary Table 1). Cytologic smears were prepared from tumor samples by a board-certified cytopathologist (KS) who evaluated the tumor content using a standard benchtop microscope (Olympus). Next, specimens were analyzed with the EpiView-D4, in similar fashion to the animal experiments. The investigator team was un-blinded to HER2 status only after testing of all specimens was complete.

**Fig. 5:**
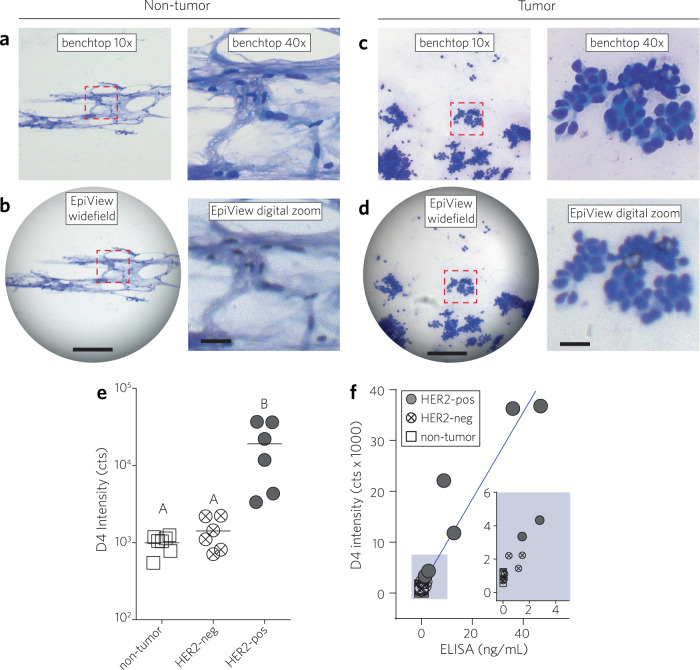
Evaluation of tissues obtained from human patients with EpiView-D4. Brightfield cytology imaging of non-tumor human breast tissue specimens were sampled by FNA and imaged by standard benchtop microscopy (**a**) and brightfield imaging by EpiView (**b**). Left panels: LPF views by standard microscope equipped with a 10× objective lens (top) and native view on EpiView (bottom). Right panels: standard microscope image obtained by a 40× objective (top), showing the same ROI outlined with red dashed line in the 10× image, and a similar view obtained by digital zoom on the EpiView (bottom). Scale bars for LPF and HPF images are 0.2 mm and 30 µm, respectively. **c**, **d** Similar sequence of images as **a** and **b** but with a representative specimen with cytological evidence of tumor. **e** Results of HER2 credentialing by EpiView-D4 for 19 different patient breast tissue specimens: non-tumor, HER2-negative tumor, HER2-positive tumor (*N* = 7, 6, 6, respectively). Each data point represents the mean D4 fluorescence intensity (after background subtraction) of duplicate assays measured by the EpiView-D4 for an individual tissue specimen. The horizontal line represents the mean D4 signal intensity for each category. There was a statistically significant difference between groups as determined by one-way ANOVA (F (2, 16) = 9.264, *p* = 0.0021). Groups that are different by multiple comparison testing (Tukey post hoc test, *p* ≤ 0.05) are marked with different letters. HER2-positive tumors are defined as IHC 3+ or IHC 2+ with HER2 amplification by FISH. **f** EpiView-D4 readout vs ELISA for HER2 for all patient specimens. A strong, positive correlation between EpiView-D4 and ELISA across the 19 paired measurements is observed, which was statistically significant (Pearson *r* = 0.956, *p* < 0.0001, 95% CI: 0.886–0.983).

A summary of the clinical pathology characteristics of each patient specimen is shown in Supplementary Table 1. Review of FNA cytologic preparations identified tumor cells in 12 specimens, while no tumor cells — and only fibroadipose tissue— were identified in the remaining 7 specimens. Fig. 5a–d show representative brightfield LPF and HPF images of FNA cytology preparations recorded on both the EpiView-D4 and a standard upright microscope for both non-tumor (Fig. 5a, b) and tumor (Fig. 5c, d) specimens. As with the mouse xenograft samples, brightfield images taken by the EpiView-D4 were of sufficient quality to confirm cellularity of the sample. Of the 12 tumor specimens identified, review of IHC and follow-up fluorescence in situ hybridization (FISH) at or near the time of resection revealed that 6 specimens were known to be clinically HER2-positive and 6 specimens were HER2-negative. Fig. 5e shows the D4 fluorescence intensity for all 19 specimens, segregated into 3 groups: fibroadipose (non-tumor) breast tissue (*N* = 7), HER2-negative tumors (*N* = 6), and HER2-positive tumors (*N* = 6). There was a statistically significant difference in the D4 fluorescence signal between the groups, as determined by one-way ANOVA (F (2, 16) = 9.264, *p* = 0.0021). Multiple comparisons by Tukey’s post hoc testing revealed that the HER2-positive group exhibited a higher fluorescence signal compared to both the HER2-negative and non-tumor groups (*p* < 0.05), and there was no statistical difference between the HER2-negative and non-tumor groups (*p* > 0.05). With 3 groups of at least 6 independent samples each, there is 81% power to detect an effect size of 0.75, where the effect size is the difference in means divided by the standard deviation. An effect size of 0.75 is considered moderate. Further, comparison of EpiView-D4 readout to HER2 ELISA (Fig. 5f), run separately by staff at the Stedman Immunoassay Lab at DUMC, showed a strong, positive correlation between EpiView-D4 and ELISA across the 19 paired measurements, which was statistically significant (Pearson *r* = 0.956, *p* < 0.0001, 95% CI: 0.886–0.983). Combined, these results highlight the potential clinical translatability of the EpiView-D4 for cytopathological evaluation of breast cancer and discriminating HER2-positive from HER2-negative tumors using FNA samples.

## Discussion

We have developed a mobile pathology platform, the EpiView-D4, that can evaluate both cytology and molecular biomarker (HER2) expression of breast tumors sampled by FNA. Our work was motivated by the need for affordable and easy to use —and disseminate—alternatives to conventional methods of diagnostic breast pathology, which currently rely on resource-intensive histological and immunohistochemical staining and interpretation of sectioned tissue samples. The experiments described herein provide proof-of-concept demonstration that our workflow —sampling, chip readout of biomarker expression, and cytology assessment—can be accomplished on a single, low-cost device in an hour or less.

In abundant resource settings, CNB is preferred over FNA, as the latter lacks information on invasion status and is associated with poorer diagnostic performance particularly with non-palpable lesions^52^. However, in limited resource settings, breast tumors most predominantly present with symptoms due to negligible access to breast cancer screening, and in these instances FNA can diagnose malignancy with excellent sensitivities and specificities of 89–98.5% and 98–100%, respectively^53,54^. Furthermore, FNA is well-suited for rural clinics as it is rapid, minimally invasive, and requires minimal infrastructure. In fact, an ongoing clinical study in Peru (PATH) is already showing success in implementing FNA for cytology assessment of breast cancer in limited-resource settings^55,56^.

The EpiView-D4 is also potentially synergistic with other innovative technologies under development that share the same goal of improving breast screening in the developing world. For example, one can envision a workflow in which initial screening is done in the field by the EpiView-D4, and cases requiring more granular assessment of tumor heterogeneity are referred to local clinics for CytoPAN or traditional cell-block pathology analysis, which provides detailed visualization and quantitation of biomarker expression at the level of individual tumor cells^35^. Other technologies that may complement the EpiView-D4 include “digital breast exam” devices that identify tissue abnormalities based on tactile imaging^57,58^, piezoelectric palpation^59,60^, or microelectromechanical sensing^61,62^, that might identify populations with less obvious lesions who may benefit from further workup with our device.

The EpiView itself is an innovative smartphone microscopy platform that combines dual functions in a single device —brightfield imaging for cytology inspection and fluorescence imaging for D4 assay quantification. The EpiView has a modular design with switchable imaging attachments to change between imaging modalities based on the following considerations: (1) it protects the optical integrity of each imaging mode to the maximum extent by keeping key optical elements within a module fixed and hence easily exchanged as a unit; (2) because of this modular design, we also gain more flexibility in the choice of optical components, as new attachments can be designed in a bespoke manner, based on need. For instance, we used two different focal length external lenses for the two imaging units in our current design to meet different application needs, which is not possible in the previous multimodal smartphone microscopy design. An additional advantage of the EpiView is its connectivity, particularly as widespread access to the internet and availability of cellular networks (even in limited-resource settings) can allow users to readily transmit digital results to remote providers offsite who can interpret results and triage cases to expedite time to diagnosis and hence adequate treatment. This is especially important in many limited resource settings where trained pathologists are scarce, with fewer than one pathologist for every million people in some countries^63^.

In the brightfield mode, we achieved a FOV of ~0.8 mm^2^ which is comparable to that achieved on a benchtop microscope with a 10× objective, while maintaining an imaging resolution of slightly over 1 µm. Combined, these characteristics translated to the ability to analyze the salient cytological features captured from FNA specimens. If an even larger FOV is required for other applications, it is important to note that the FOV can be easily expanded to a few cm^2^ by selecting a different external lens. In fluorescence mode, the EpiView platform sets itself apart from previous smartphone fluorescence microscopes by implementing a new epifluorescence illumination configuration. This design has several advantages over the tilted illumination in our previous embodiment:^47^ (1) It provides a vertical illumination and epi-detection, which maximizes the performance of the emission filter, improves background noise rejection, and therefore enhances the signal-to-noise ratio (SNR) and detection sensitivity; (2) It also brings better illumination uniformity and adjustable illumination area by incorporating a miniature beam expander; (3) It reduces the size and weight of moving parts to the sample slide itself, while in previous designs the excitation light source had to be moved together with the sample slide during focusing. Taken together, these attributes of the EpiView significantly improves the robustness and mechanical stability of the device.

We observed that transitioning to the handheld EpiView-D4 from the scanner led to a ~3.6-fold decrease in sensitivity for the D4 assay. In context, this represents a notable performance improvement compared to an older embodiment of the mobile microscope that showed a ~20-fold decrease in sensitivity compared to the tabletop scanner^36^. We attribute the improved performance observed with the EpiView-D4 to improved illumination and increased noise rejection efficiency due to the epifluorescence optical configuration. For other applications, the sensitivity of our device could be further improved by switching to a monochromic camera-embedded smartphone^64^, or combining with other signal enhancement strategies, such as surface-enhanced fluorescence^65,66^.

Several aspects of our proposed approach for biomarker assessment warrant discussion. First, the sandwich immunoassay of tumor cell lysate by the EpiView-D4 departs from standard practice of IHC, which unlike FNA preserves cellular morphology and structural relationships. However, the user-friendliness of our method and quantitative readout of the D4 assay may offer an acceptable tradeoff in limited-resource settings, especially as the workflow of traditional IHC is susceptible to user variation and subjective interpretation of results, and has a steeper learning curve^67–70^. Early work suggests that lysates analyzed by western blotting show good agreement with IHC when assessing HER2 overexpression^48^, and our results with animal and human tumor specimens which showed good concordance between western blotting and the EpiViewD4 assay are promising. However, larger-scale clinical studies will be needed to benchmark the utility and diagnostic performance of the EpiView-D4 compared to gold standard cell-block pathology methods and therefore our technology cannot currently be used as the sole method of pathological evaluation or to select targeted therapies. Rather, the EpiView-D4 may be useful as a screening or triaging tool to identify patients who should undergo more extensive clinical and pathological evaluation.

Second, analyzing biomarker expression levels in lysates from mixed samples (i.e., healthy and tumor tissue) or from tumors with heterogeneous expression of biomarkers on the D4 chip are likely to confound results. Although specimens acquired via FNA are often highly enriched in tumor cells^71^, a rudimentary workaround might be sampling many different sites and rapidly analyzing them on-the-spot (which is much easier to accomplish with FNA than CNB) to increase the likelihood of adequately representing the tumor. An alternative strategy might be to use cytology images to calculate the approximate percentage of tumor cells for each sampling event and then derive a correction factor for quantitating tumor biomarker levels, particularly as normal cells can contribute to biomarker expression levels (e.g., ER, PR) measured by the D4 immunodiagnostic assay. We expect that the latter approach will be effective, as similar methods have been used for next-generation sequencing analysis from FNA samples of solid organ malignancies^71^.

Beyond these considerations, the D4 assay platform has several design characteristics which make it advantageous for use in limited-resource settings. The D4 contains all necessary assay reagents “on-chip” and automatically runs to completion upon addition of a biological fluid (such as crude tumor cell lysate as shown here) with no additional reagents required, which minimizes user intervention. Furthermore, as the assay reagents are printed on a stabilizing polymer brush surface, assays can be stored without refrigeration, which is often unavailable in limited-resource settings. Additionally, manufacturing by inkjet printing is straightforward and easily scalable, which can expedite distribution to areas of need. D4 chips are also low-cost; the highest cost of immunoassays usually stems from the cost of antibody reagents, and the D4 only utilizes about 5 nanograms of cAb and 50 nanograms of dAb per assay. Finally, when read by a portable detector, such as the EpiView, D4 chips could be deployed and operated in remote environments.

There are also several limitations to this study. First, results produced by our device still requires remote analysis of data uploaded to the cloud; this could be problematic in areas without cell phone signal or if trained readers are unavailable. Work by others has already generated elegant computational methods for visualizing and digitally processing pathology specimens^72–77^. A comprehensive review of these methods is outside the scope of this paper, but the concept of fully automating analysis and readout with a user-friendly, on-board “app” that incorporates deep learning and related techniques —which have already been shown to be very powerful in medical imaging^78–82^— would be a powerful way to further reduce infrastructure and user-dependencies. We are actively developing machine learning algorithms to accomplish automated interpretation of cytology preparations and readout of D4 fluorescence on the EpiView-D4. Second, a key bottleneck in our current approach is that we determined the mass of each FNA specimen prior to D4 analysis to ensure lysates had similar concentration (mass per volume) of tissue for comparison. This step is not practical for clinical or limited resource settings. In the short term, this could be addressed by engineering methods to (1) standardize the amount of tissue obtained at the aspiration step, or (2) use loading controls (as in western blotting) to normalize the readout.

Third, the D4 chip used here focused solely on the measurement of HER2. To adequately profile breast tumors for clinical use, the chip must include multiplexed quantitation of ER, PR, and Ki67 as well. Fortunately, this issue is easy to address, as we have previously shown that as long as non-cross reactive antibody pairs exist for these targets, fabricating multiplexed D4 assays is relatively straightforward^36^. Fourth, we used D4 chips with a similar architecture to those used in our prior work that require a single wash step after incubation^36^. While easy to perform, we would prefer to eliminate this single wash step to make the D4 workflow truly intervention-free. To address this limitation, we are completing the development of an enclosed, passively driven microfluidic design for our D4 chips, which will be reported separately^83^.

Fifth, it is worth noting that our studies used a limited number of fresh-frozen animal and patient tumors that were previously collected and archived by the investigators. We elected to use a curated set of frozen specimens as proof-of-concept due to their accessibility. Future work in a real-world clinical setting will be required to assess its clinical utility for LRS. Finally, although the EpiView-D4 was able to distinguish clinically HER2-positive specimens from HER2-negative and non-tumor specimens in this study, work remains to be done with larger sample sizes in order to adequately calculate the clinical sensitivity/specificity and to establish appropriate diagnostic cut-off points based on receiver-operator characteristics.

To conclude, the EpiView-D4 offers technology that can supplement existing breast cancer pathology services that are available in limited resource settings, and has the potential to democratize access to effective breast cancer treatment worldwide. While this study focused on analyzing FNA of breast tumors, it offers great flexibility for use in a variety of pathology applications as it effectively functions as a mobile brightfield and fluorescence microscope. Furthermore, because D4 chips can target any analyte for which good Ab pairs are available, the strategy described herein can be extended to other tissue types and diseases that require concurrent tissue typing and molecular analysis. Finally, beyond its relevance to patient care, our portable platform is broadly useful for laboratory research, clinical trials, and epidemiology.

## Methods

### Study design & statistical analysis

This study was designed to provide both analytical and clinicopathologic evaluation of our assay technology. We began by using recombinant protein spiked samples, and then completed in vitro (cell lines) and in vivo (animal studies) studies, followed by clinical validation in patients. For morphologic analysis of cancer cells, FNA was specifically chosen for sampling in this study as it is the preferred method for cellular pathology in limited resource settings. Sample sizes and *p* values are indicated in the text and/or figure legends, wherein *p* < 0.05 was considered significant.

For analytical studies, we utilized spiked samples in duplicates. The limit-of-blank (LOB) and limit-of-detection (LOD) were calculated according to Armbruster and Pry^84^. Data were fit to a five-parameter logistic (5-PL) fit curve using OriginPro 9.0 (OriginLab Corp.). For the in vitro studies using established breast cancer cell lines (Fig. 2e), we performed experiments utilizing at least 6 replicates. For animal studies, 16 specimens were collected from three different xenograft models in nude mice: BT474, MDA-MB-453, or BT20 (*N* = 8, 3, 5, respectively). The xenograft studies were not blinded. Univariate statistical analysis was performed to compare the HER2 fluorescence intensity between groups using a one-way ANOVA, followed by Tukey’s post-hoc test (GraphPad Prism 6). Samples were tested independently via ELISA in duplicate by the clinical laboratory at DUMC. To assess the correlation between ELISA and D4 readout, Pearson’s r correlation was calculated (GraphPad Prism 6).

The human studies were performed with previously collected tumor samples that were resected as part of routine clinical care for invasive breast cancer; informed consent was obtained from all human participants. Tumor samples were collected from excess tissue not required for pathologic diagnosis, then banked at −80 °C in accordance with an IRB-approved prospective tumor repository. Twenty-one consecutive de-identified samples that were of sufficient size to permit FNA aspiration were selected (Duke IRB Protocol Pro00100360). The only clinical data obtained from the tumor registry was baseline ER, PR, and HER2; two samples had conflicting or missing pathology documentation in the medical record, and these samples were hence excluded. We oversampled for HER2-positive status. Overall, 19 cases were collected and analyzed, 6 of which were confirmed to be HER2-positive, 6 HER2-negative, and 7 fibroadipose breast tissue. HER2 status was not known to the investigator performing the EpiView-D4 analysis or the staff performing ELISA. Univariate statistical analysis was performed to compare the HER2 fluorescence intensity between groups using a one-way ANOVA, followed by Tukey’s post-hoc test (GraphPad Prism 6). With 3 groups comprised of 6 independent samples each, there is 81% power to detect an effect size 0f 0.75, where the effect size is the difference in means divided by the standard deviation. An effect size of 0.75 is considered moderate. Correlation between ELISA and D4 readout was assessed using Pearson’s r correlation (GraphPad Prism 6).

### Design and preparation of the EpiView scope

The dual function smartphone microscope (EpiView) consists of 3D-printed optomechanical attachments and a Nokia Lumia 1020 smartphone. The 3D-printed attachments were designed on Autodesk Inventor and fabricated by 3D printing (StrataSys uPrint SE plus). The construct includes a base attachment that is mounted directly on the smartphone and two switchable imaging imaging modules for brightfield and epifluorescence imaging. Both imaging modules can be slid into the base attachment to form a standalone handheld device for brightfield or epifluorescence imaging applications. In the assembled brightfield device, a 1/2” dovetail translation focusing stage (DT12, Thorlabs) is mounted above the external lens for focusing adjustment. The imaging module contains a white LED (3.2 V, 897–1183-ND, Digikey) powered by a coin cell battery (CR2032) for illumination, a glass slide sample holder, and two pieces of filter paper as diffuser placed between the light source and the sample. A lens module (f_2_ = 2.6 mm, UCTronics) is placed in front of the smartphone camera as the objective. The smartphone has a 2/3-inch, 38 megapixels (5360×7152) complementary metal-oxide semiconductor (CMOS) image sensor. The lens on the smartphone camera has a focal length of f_1_ = 6.86 mm, so that the magnification of the brightfield imaging system, M = f_1_/f_2_ ≈ 2.6×.

In the epifluorescence attachment, a green laser diode (532 nm, <150 mW, Z-bolt) powered by 2 AA batteries is connected to a heatsink and mounted together on the base attachment. The laser beam is expanded by a beam-expander module comprising a concave lens (f_3_ = −9 mm, #84–378, Edmund Optics) and a convex lens (f_4_ = 24 mm, #65–480, Edmund Optics) which together give a beam magnifying power MF = −f_4_/f_3_ ≈ 2.6×. After passing the beam focusing lens (f_5_ = 15 mm, #69–387, Edmund Optics) the beam is deflected by 90° using a dichroic mirror (#34–736, Edmund Optics) with the cut-on wavelength of 552 nm. The beam is focused again by an objective lens module (f_6_ ≈ 8 mm, DTR’s laser shop) before reaching the sample. A 585 nm band-pass filter (#33–906) is placed in front of the smartphone camera as an emission filter to collect fluorescence from the sample. A micro-focusing stage and a sample holder are also placed in the imaging module similar to the brightfield attachment. A white LED (3.2 V, 897–1183-ND, Digikey) powered with cell battery is used as the light source for pre-focusing.

### Image acquisition with EpiView and data analysis

Brightfield imaging was performed on the EpiView to capture the images of both Diff-Quik^TM^ and HER2-IHC slides. To do so, a sample slide was inserted to the sample holder of the brightfield imaging module, followed by turning on LED and focusing. The built-in smartphone camera app (Nokia Camera Pro) was used for digital image acquisition. An integration time of 0.4 s and an ISO value of 200 was used for both Diff-Quik^TM^ and HER2-IHC slide. All the images were stored as lossless raw format (DNG file), which can be converted to TIFF file for further analysis with ImageJ or MATLAB.

To test the spatial resolution of EpiView, an USAF 1951 resolution test target was imaged with brightfield and epifluorescence imaging attachments (in brightfield mode), respectively. The resolution target was place between the white LED and the smartphone camera in both imaging attachment modules. The Smartphone imaging of fluorescent microbeads was performed on EpiView to quantify the sensitivity of EpiView. Red polystyrene microspheres (FluoSpheres™, 580 nm/605 nm, ThermoFischer) with diameters ranging from 1 μm to 200 nm were used. A suspension of fluorescent microspheres was diluted 5000–1,000,000 times depending on the size of the microsphere for desired density of microspheres in a field of view. The glass coverslip was rinsed with acetone, isopropanol, and methanol, blow-dried, and finally treated with plasma for a few seconds. 1 μL diluted microsphere suspension was added to the coverslip and dried in air. The sample was then inserted into the coverslip holder of the fluorescence module for imaging. The white LED was first used to focus the sample before the laser was turned on to minimize photobleaching and laser overheating. An integration time of 2 s and an ISO value of 400 was used in the imaging of 100 nm microspheres. To validate single nanoparticle imaging, the same fluorescent nanoparticle sample used in the sensitivity test was also imaged by a benchtop inverted microscope (Olympus IX83). The fluorescent sample was imaged by using a 100× oil immersion objective lens (NA = 1.40) of the same area imaged by the smartphone microscope. Digital images were acquired in Cy3 channel with an integration time of 1 s by using a scientific CMOS camera (Hamamatsu Flash4.0).

A similar procedure was used for fluorescence imaging of the D4 chips. An integration time of 1 s and an ISO value of 400 was used in imaging all D4 samples. To quantify the fluorescence intensity of D4 microarrays, the raw smartphone DNG images were first converted to RGB TIFF images. A monochromic TIFF image was generated by averaging the values of each of the red (R), green (G) and blue (B) channels. The monochromic image was analyzed using ImageJ.

### Brightfield image deconvolution

Brightfield image deconvolution was conducted using the Lucy-Richardson^85^ algorithm in MATLAB. Before starting the deconvolution, the point spread function (PSF) of the smartphone microscope was measured by imaging single 500-nm polystyrene microbeads in brightfield mode. To obtain the PSF image, 20 single microbeads were averaged. The averaged image of a single microbead was then split into red (R), green (G) and blue (B) channels (Supplementary Fig. 1). The central 2 by 2 pixels were used as the PSF of each color channel during the deconvolution process. To deconvolve raw images, the original smartphone brightfield images were also split into R, G and B channels. Then, each channel was deconvoluted separately with the corresponding PSF of that channel. The deconvoluted single channel images were then merged in ImageJ to generate the deconvoluted RGB image with enhanced resolution and contrast. The intensity value of each deconvoluted color channel was manually adjusted in ImageJ to compensate the color difference before and after deconvolution.

### D4 immunoassay fabrication

Details regarding the fabrication and characterization of D4 immunoassays have been described extensively elsewhere^36^. In brief, POEGMA polymer brush coatings were “grafted from” glass slides via surface-initiated atom transfer radical polymerization as described previously^36^. HER2 capture/detection Ab pairs (cat# MAB1129/AF1129, respectively, R&D Systems, Inc.) were then deposited onto POEGMA-coated slides as follows. The cAbs (4 mg/mL in 1x PBS) were printed onto POEGMA-coated substrates with a Scienion S11 sciFLEXARRAYER (Scienion AG). Next, twelve 100 nL trehalose pads (10% (w/v) trehalose solution in 0.2 µm filtered DI water) were printed around the periphery of the cAb array using a BioDot AD1520 printer (BioDot, Inc.); these pads formed a soluble underlayer onto which dAbs (conjugated to AlexaFluor 532 or AlexaFluor 647 fluorophores per the manufacturer’s instructions) would be printed. The dAb printing ink was prepared by dissolving fluorophore-tagged dAb at 0.05 mg/mL in a solution of 5% (w/v) trehalose in 1× PBS; this ink was then deposited on top of the trehalose pads with the BioDot AD1520 printer. After printing, D4 chips were vacuum desiccated (30 kPa) to facilitate cAb immobilization into the POEGMA brush, and then stored at room temperature for later use.

### D4 immunoassay

To determine analytical sensitivity, analyte binding curves were generated using recombinant HER2 protein (R&D systems, Inc.) spiked into RIPA buffer. D4 chips were incubated with a dilution series of analyte spiked into RIPA buffer (Thermo Scientific^TM^) for 60 min. Substrate were then briefly rinsed in 0.1% Tween-20/PBS, and then dried. D4 chips were imaged on an Axon Genepix 4400 tabletop scanner (Molecular Devices, LLC) and the custom EpiView device (as described above). The limit-of-blank (LOB) and limit-of-detection (LOD) were calculated according to Armbruster and Pry^84^: in brief, mean fluorescence intensity (µ) and SD (σ) from vehicle samples were used to calculate LOB = µ_blank_ + 1.645σ_blank_. LOD was then estimated from spiked low-concentration samples (LCS) above the LOB, such that LOD = LOB + 1.645σ_LCS_. Data were fit to a five-parameter logistic (5-PL) fit curve using OriginPro 9.0 (OriginLab Corp.).

For evaluation of HER2 expression for in vitro studies, human breast cancer cell lines were harvested and resuspended in RIPA buffer at a density of 20,000 cells per μL. 50 µL samples of crude cell lysate were incubated on the D4 chip for 60 min. After incubation, the chips were briefly rinsed in a 0.1% Tween-20/PBS wash buffer then dried. Chips were imaged using the Axon Genepix 4400 scanner.

For evaluation of HER2 expression in solid tumors (animals and human patient), tissue specimens obtained by FNA were mixed with RIPA buffer at a density of 1 mg per 100 µL (xenografts) or 1 mg per 200 µL (patient samples). 50 µL of each lysate was added to the D4 chip in duplicate, and samples were incubated for 60 min, followed by a wash/dry step as before. All D4 chips were imaged with both the EpiView-D4 device and the Genepix 4400 scanner for comparison.

### Enzyme-linked immunosorbent assay (ELISA)

ELISA was performed independently by staff members at the Stedman Immunoassay Laboratory at DUMC on a fee-for-service basis. The HER2 concentration in cell culture and tumor lysate (processed with RIPA buffer and diluted 5–100×) were measured in duplicate using a human ErbB2/Her2 ELISA kit according to the manufacturer’s instruction (R&D Systems, Inc.).

### Cell culture

All established breast cancer cell lines (BT474, BT20, MDA-MB-231, MDA-MB-453, MDA-MB-468) were obtained from the Cell Culture Facility at Duke University and tested by the facility for mycoplasma contamination and cell line identity. Cells were cultured in DMEM with 10% FBS and maintained at 37 °C in 5% CO_2_. For primary patient-derived tumor cell lines, breast tumor samples were obtained from the Duke Biospecimen Repository and Processing Core (BRPC) under Duke University IRB protocol Pro00066580. Samples were digested and conditionally re-programmed as previously described elsewhere^46^. Briefly, tumors were digested and plated on top of irradiated fibroblasts in the presence of a ROCK inhibitor. After 7–10 days, fibroblasts were trypsinized and removed, leaving a pure population of breast cancer cells.

### Western blotting and antibodies

Lysates were made by resuspending cells in NuPage Sample Buffer, incubated on ice for 15 min, then clarified at 13,000 RPM, 4 °C, for 10 min. Protein content was quantified using the Bradford method. Immunoblotting was performed as previously described^45^ and membranes were probed with primary antibodies (1:1,000 dilution) recognizing vinculin (CST#4650), H3 (CST#4499), β-Actin (CST#4970), and HER2 (CST#4290). For all representative immunoblots in the manuscript, experiments were conducted at least twice, had no repeatability issues. All blots derive from the same experiment and were processed in parallel. Un-cropped, raw images of Western blots are provided in Supplementary Figs 5 and 6.

### Genome editing of breast cancer cell lines

We tested the specificity of the HER2 D4 assay in vitro by modulating HER2 expression and evaluating its correlation to D4 fluorescence signal. We used CRISPR genome editing to knockout HER2 expression in HER2+ tumor cells and insertion of an ORF that encodes HER2 to upregulate HER2 expression in HER2- tumor cells. The following sections detail the reagents/methods necessary for this experiment: Cloning CRISPR constructs, obtaining the ORF construct, producing lentivirus, and transducing mammalian cells.

#### Cloning CRISPR constructs

CRISPR constructs were cloned following an established and field standard method^86^ using previously characterized sgRNAs^87^. sgRNA inserts were synthesized by IDT as follows:

GGAAAGGACGAAACACCGXXXXXXXXXXXXXXXXXXXXGTTTTAGAGCTAGAAATAGCAAGTTAAAATAAGGC

”X” denotes a unique 20mer sgRNA sequence

The oligo pool was diluted 1:100 in water and amplified using NEB Phusion Hotstart Flex enzyme master mix and the following primers:

Forward:

TAACTTGAAAGTATTTCGATTTCTTGGCTTTATATATCTTGTGGAAAGGACGAAACACCG

Reverse:

ACTTTTTCAAGTTGATAACGGACTAGCCTTATTTTAACTTGCTATTTCTAGCTCTAAAAC

PCR Protocol: 98 °C/30 s, 18×[98 °C/10 s, 63 °C/10 s, 72 °C/15 s], 72 °C/3 min

Inserts were purified with Axygen PCR clean-up beads (1.8x; Fisher Scientific) and resuspended in molecular biology grade water. lentiCRISPRv2 (hygro) was digested with BsmBI (Thermo Fisher) for 2 h at 37 °C. The large ~13kB band was gel extracted after size-selection on a 1% agarose gel. Using 100 ng of cut lentiCRISPRv2 and 40 ng of sgRNA oligos, a 20 μL Gibson assembly reaction was performed (30 min, 50 °C). After Gibson assembly, 1 μL of the reaction was transformed into electrocompetent Lucigen cells and spread on LB-ampicillin plates and incubated overnight. Single colonies were picked and underwent plasmid extraction using a Plasmid miniprep kit (Qiagen). Following plasmid DNA extraction, lentivirus was made for each construct following the protocol below.

23-mer Sequences:

sg ctrl: GTAGCGAACGTGTCCGGCGT

sgHER2 #1: GCACCGTGTGCACGAAGCAG

sgHER2 #2: GAGACTGCTGCAGGAAACGG

sgHER2 #3: ACAATCCGCAGCCTCTGCAG

#### Preparing ORF constructs

Constructs were purchased, lentivirus was produced for each construct and used to infect target cells as previously described. X4 (Luciferase) sequence information can be found in the literature^88^. The HER2 ORF was obtained from genecopoeia (clone ID# EX-B0017-Lv105).

#### Lentiviral production and transduction

HEK 293FT cells (obtained from the Cell Culture Facility at Duke University) were grown in 10 cm diameter plates to ~90% confluence. For each plate, transfection was performed using Lipofectamine 2000 (Life Technologies), 8.18 μg of psPAX2, 5.34 μg pVSVg, and 10.7 μg of CRISPR or ORF plasmid. After 5 min of incubation at room temperature, the mixture was added to the cells and incubated overnight. After 18 h, harvest media was added (DMEM 30% FBS). After one 48 h collection, the harvested virus was passed through a 0.45 μm filter. Transductions were performed by seeding cells at ~40% confluence into 6-well dishes, then the following day 0.5 mL of virus, 0.5 mL of media, and 2 μL polybrene were added to the cells. The cells were then centrifuged at 2250 RPM for 1 h at 25 °C. Following centrifugation, the cells were incubated with fresh media. The following day, cells were selected with an appropriate selection antibiotic.

### Fine-needle aspiration and cytopathology specimen preparation

All cytopathology specimen preparation was performed by a clinical cytopathologist. Solid tumor specimens (from animal models or human patients) were sampled by the Zajdela^51^ (capillary action) FNA technique using 23-gauge needles. Aspirates were smeared onto standard positively charged microscope slides. Specimens were stained using a commercially available Diff-Quik^TM^ rapid staining kit (Polysciences, Inc.), according to the manufacturer’s protocol. After staining, slides were imaged using a conventional brightfield microscope at 10× and 40× magnification for low- and high-power field imaging. Slides were also imaged using the EpiView-D4 device as described above. All cytology smears were formally interpreted by a pathologist.

### Orthotopic xenograft tumor studies

All procedures performed were approved by the Institutional Animal Care and Use Committee at Duke University. Cultured BT474, MDA-MB-453, or BT20 breast cancer cells were trypsinized at 80% confluence, washed with PBS, and resuspended in a 1:1 mixture of serum free medium and Matrigel (Corning Inc.). The cells (1–5 × 10^6^) were then orthotopically implanted into the axial mammary fat pads of 6-week old *Nu/Nu* mice (The Jackson Laboratory). Of note, 48 h prior to the injection of cells, mice receiving BT474 tumors were ovariectomized and implanted with estradiol (0.72 mg/60 days continuous release) treatment pellets (Innovative Research of America) to facilitate tumor initiation. Tumors dimensions were measured three times weekly with calipers, and body weight and behavior were assessed at the time of measurement. Tumor volume was calculated as A × B2 × 0.5, where A is the longer of the perpendicular axes. After reaching 0.5–1 cm^3^ volume (6–10 weeks depending on cell line), tumors were excised following humane euthanasia, cryopreserved and archived at -80 °C for later use.

### Immunohistochemistry of xenografts

All IHC staining of tumor xenografts was performed by the Duke Research Immunohistology Laboratory on a fee-for-service basis. Harvested tumor tissue was fixed in 2% neutral buffered formalin and embedded in paraffin. Thin, ~5 µm tissue slices were cut using a microtome, and the sections were mounted for antigen retrieval. Standard immunohistochemistry methods of deparaffinizing and rehydrating with solvents were used, followed by immunostaining of tissue sections with primary antibody against HER2 (Abcam cat# ab214275) at 1:100 dilution for 45 min at room temperature and then labeling with HRP-conjugated secondary detection antibody for 30 min at room temperature and detected with Vector’s RTU Elite ABC reagent (Cat# PK-7100) for 30 min at room temperature. Underlying tissue structures were visualized by counterstaining with Harris hematoxylin.

### Use of human breast tumor specimens

19 breast tissue specimens were collected as part of this study. The study was approved by the Duke IRB (Pro00100360), and informed consent was obtained from all human participants. Human breast tumor specimens were collected by the breast surgical oncology group at DUMC at the time of definitive surgery and stored as fresh-frozen specimens at −80 °C prior to analysis. IHC for HER2 receptor status was performed preoperatively in a CLIA certified clinical lab for all samples included in this study. The specimens were provided to the investigator team in a de-identified and blinded fashion. These were analyzed by the for cytopathology and HER2 levels as described in greater detail in the “Study Design” above.

### Reporting summary

Further information on research design is available in the Nature Research Reporting Summary linked to this article.

## Supplementary information

Supplementary Information

Reporting Summary

## Data Availability

The data generated and analyzed during this study are described in the following data record: 10.6084/m9.figshare.14703405^89^. The majority of data files are shared openly together with the data record. However, 4 files have not been shared publicly as they contain private patient health information and permission to share these data was not obtained. Parties wishing to request access to these data files should contact the corresponding author. A detailed list of which data file underlies which element of the article, along with their availability, is included with the data record in the file ‘Joh_et_al_2021_underlying_data_files_list.xlsx’.

## Code availability

Code used for brightfield image deconvolution (Lucy-Richardson^85^ algorithm) is commercially available in MATLAB (R2019b) software. Additional software used for the study are GenePix Pro 7, Graphpad Prism 6, OriginPro 9, ImageJ 1.51j8, all of which are commercially or publicly available.
