# Author Correction: Cellphone enabled point-of-care assessment of breast tumor cytology and molecular HER2 expression from fine-needle aspirates

**DOI:** 10.1038/s41523-021-00335-4

**Published:** 2021-09-17

**Authors:** Daniel Y. Joh, Jacob T. Heggestad, Shengwei Zhang, Gray R. Anderson, Jayanta Bhattacharyya, Suzanne E. Wardell, Simone A. Wall, Amy B. Cheng, Faris Albarghouthi, Jason Liu, Sachi Oshima, Angus M. Hucknall, Terry Hyslop, Allison H. S. Hall, Kris C. Wood, E. Shelley Hwang, Kyle C. Strickland, Qingshan Wei, Ashutosh Chilkoti

**Affiliations:** 1 Department of Biomedical Engineering, Pratt School of Engineering, Duke University, Durham, NC USA; 2 Division of Plastic, Maxillofacial, and Oral Surgery, Department of Surgery, Duke University Medical Center, Durham, NC USA; 3 Department of Chemical and Biomolecular Engineering, North Carolina State University, Raleigh, NC USA; 4 Department of Pharmacology and Cancer Biology, Duke University School of Medicine, Durham, NC USA; 5 Center for Biomedical Engineering, Indian Institute of Technology, Hauz Khas, New Delhi, 110016 India; 6 Division of Surgical Oncology, Department of Surgery, Duke University Medical Center, Durham, NC USA; 7 Departmment of Biostatistics & Bioinformatics, Duke University Medical Center, Durham, NC USA; 8 Department of Pathology, Duke University Medical Center, Durham, NC USA

**Keywords:** Diagnostic markers, Pathology, Translational research

Correction to: *npj Breast Cancer* 10.1038/s41523-021-00290-0, published online 02 July 2021

The original version of this Article contained errors in the author affiliations. Dr. Qingshan Wei should be listed with affiliation 3 and affiliation 9 should be removed entirely as it appears to be a duplication of affiliation 2. This has now been corrected in both the PDF and HTML versions of the Article.

