# Supplementary material for: Cellphone enabled point-of-care assessment of breast tumor cytology and molecular HER2 expression from fine-needle aspirates
Source: NPJ Breast Cancer. 2021 Jul 2;7:85. doi: 10.1038/s41523-021-00290-0 (PMC8253731; doi:10.1038/s41523-021-00290-0)
Supplement: Supplementary file 1 — Supplementary Information [file 41523_2021_290_MOESM1_ESM.pdf]

Supplementary Information for

Cellphone enabled point-of-care assessment of breast tumor cytology and molecular HER2 expression from fine-needle aspirates

Authors: Daniel Y. Joh[1,2†], Jacob T. Heggestad[1†], Shengwei Zhang[3†], Gray R. Anderson[4], Jayanta Bhattacharyya[5], Suzanne E. Wardell[4], Simone A. Wall[1], Amy B. Cheng[1], Faris Albarghouthi[1], Jason Liu[1], Sachi Oshima[6], Angus M. Hucknall[1], Terry Hyslop[7], Allison H.S. Hall[8], Kris C. Wood[4], E. Shelley Hwang[6], Kyle C. Strickland[8], Qingshan Wei[2*], Ashutosh Chilkoti[1*].

Affiliations:

[1]Department of Biomedical Engineering, Pratt School of Engineering, Duke University, Durham NC 27708 USA.

[2]Division of Plastic, Maxillofacial, and Oral Surgery, Department of Surgery, Duke University Medical Center, Durham NC 27710 USA

[3]Department of Chemical and Biomolecular Engineering, North Carolina State University, Raleigh NC 27695 USA.

[4]Department of Pharmacology and Cancer Biology, Duke University School of Medicine, Durham NC 27710 USA

[5]Center for Biomedical Engineering, Indian Institute of Technology Delhi, New Delhi, India

[6]Division of Surgical Oncology, Department of Surgery, Duke University Medical Center, Durham NC 27710 USA

[7]Departmment of Biostatistics & Bioinformatics, Duke University Medical Center, Durham NC 27710 USA

[8]Department of Pathology, Duke University Medical Center, Durham NC 27710 USA

*To whom correspondence should be addressed: Ashutosh Chilkoti (chilkoti@duke.edu) or Qingshan Wei (Qingshan Wei (qwei3@ncsu.edu)

†These authors contributed equally to this work.

Contents

1) Specificity evaluation of D4 assays against HER2 by CRISPR/Cas9 genome editing and evaluation of matrix effects (Supplementary Fig. 1)

2) Resolution enhancement of brightfield imaging by point spread function deconvolution (Supplementary Fig. 2)

3) Imaging 100 nm fluorescent beads with EpiView vs. benchtop microscope (100x objective). (Supplementary Fig. 3)

4) Comparison of standard microscopy vs. EpiView-D4 for brightfield imaging of standard IHC and cytology preparations of BT474, MDA-MB-453, and BT20 solid tumor xenografts (Supplementary Fig. 4)

5) Clinical pathology of patient specimens (Supplementary Table 1)

6) Un-cropped, raw images of Western blots (Supplementary Fig. 5, Supplementary Fig. 6)

1) Specificity evaluation of D4 assays against HER2

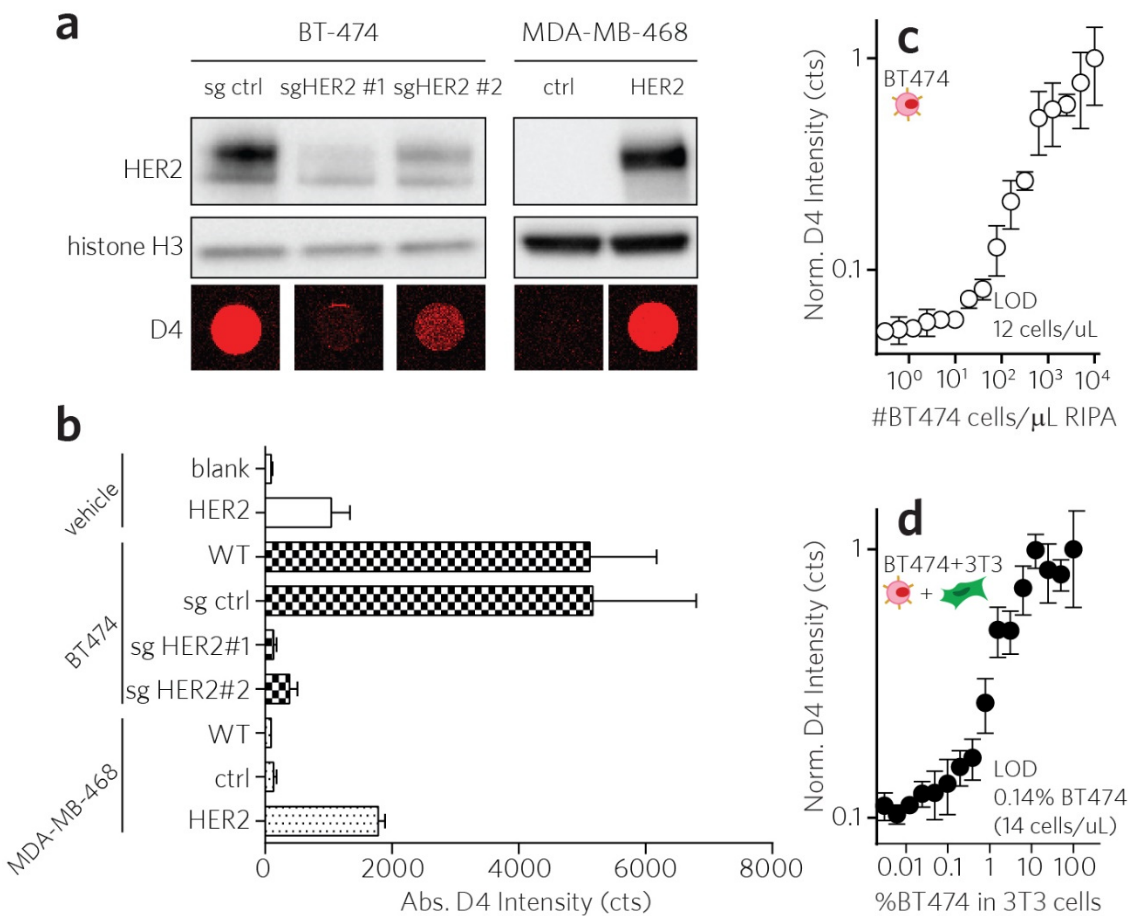

Supplementary Fig. 1 | Specificity evaluation of D4 assays against HER2 by CRISPR/Cas9 genome editing and evaluation of matrix effects. a, b, Knockdown and over-expression of HER2 by genome editing. (a) Representative western blots (top) and D4 fluorescence images (bottom row) assessing HER2 levels in pools of BT474 (left) and MDA-MB-468 (right) cells whose HER2 expression levels were modified by CRISPR/Cas9 (Histone H3 was used as a loading control). Pools of BT474 cells underwent HER2 knockdown by two independent sgRNAs (#1-2), as compared to a control pool (sg ctrl) targeting luciferase. MDA-MB-468 cells underwent stable overexpression of HER2, as compared to a control pool targeting luciferase. (b) Bar graphs quantitating D4 fluorescence intensity of HER2 signal (WT = wild type). Data represent mean ± s.d of D4 fluorescence intensity for duplicate assays. **c, d,** Evaluation of matrix effects due to introduction of fibroblast (NIH 3T3) cell lines in HER2-D4 assays, tested against BT474 cells. Normalized D4 intensity for HER2 signal plotted against dilution series of BT474 cell lysates in RIPA buffer (represented as #BT474 cells/μL) is shown in (c). Panel (d) shows similar binding curve for HER2, except 3T3 cells are introduced, and the total number of cells was kept constant (10,000 cells/μL) and the HER2 D4 signal is measured as we varied the ratio of BT474 to 3T3 cells (from 1:0 through 0:1). Data represent mean ± s.d of D4 fluorescence intensity for duplicate assays. LOD = limit of detection.

2) Resolution enhancement of brightfield imaging

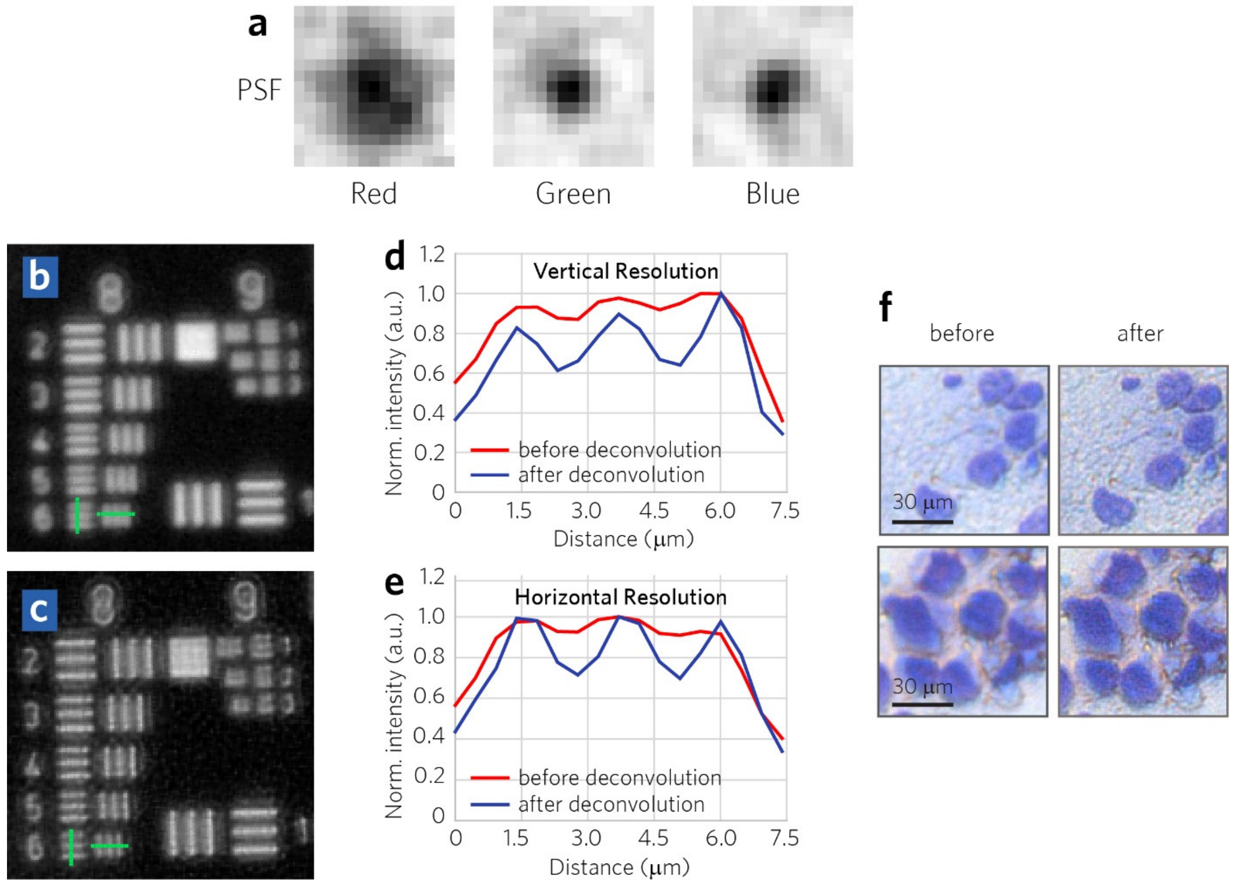

Supplementary Fig. 2 | Resolution enhancement of brightfield imaging by point spread function deconvolution. a, Image representations of point spread functions (PSF) of red, green, and blue channels in brightfield mode. **b, c,** Testing spatial resolution using USAF 1951 test target, before (b) and after (c) deconvolution with PSF. **d,** Intensity profile before (red trace) and after (blue trace) deconvolution in the vertical direction, as marked by the vertical green lines in (b,c). **e,** Intensity profile before (red trace) and after (blue trace) deconvolution in the horizontal direction, as marked by the horizontal green lines in (b,c). **f,** Representative smartphone images of FNA cytology prepared from solid tumor xenografts, before (left column) and after (right column) deconvolution.

3) Imaging 100 nm fluorescent beads

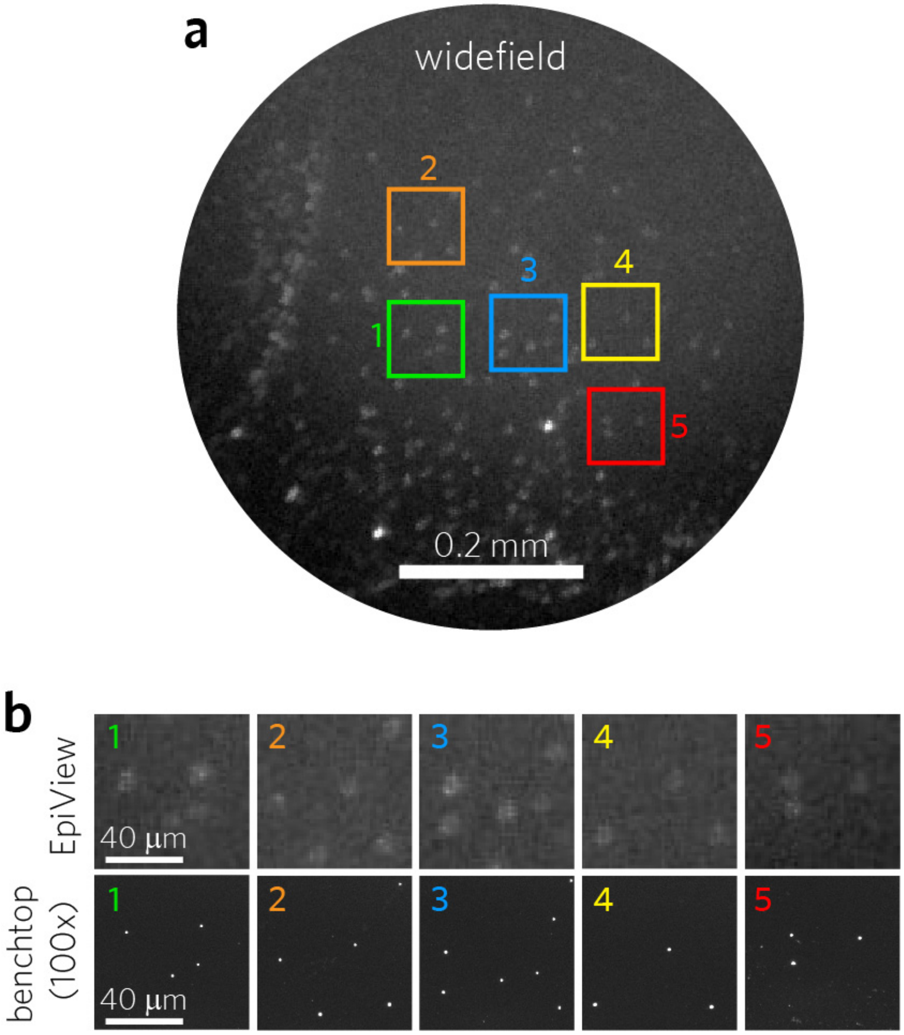

Supplementary Fig. 3 | Imaging 100 nm fluorescent beads with EpiView vs. benchtop microscope (100x objective). a, Widefield view of 100 nm fluorescent beads using EpiView in epifluorescence mode. **b,** Comparison of EpiView versus benchtop microscope (100x objective, NA = 1.4) of the 100 nm beads outlined by the numbered ROIs in the widefield image. Images were extracted from green channels.

4) Comparison of standard microscopy vs. EpiView-D4 for brightfield imaging of standard IHC and cytology preparations of tumor xenografts

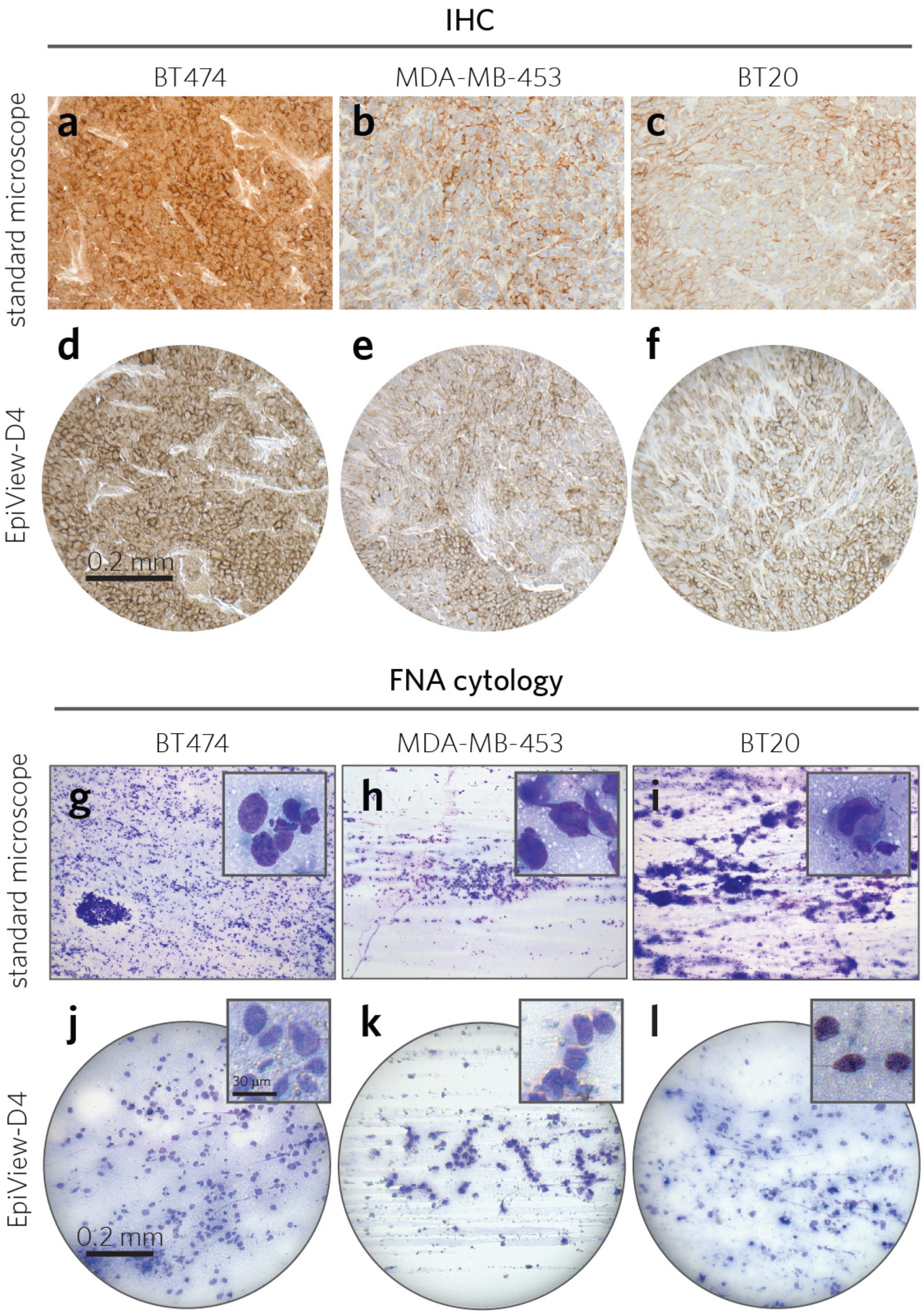

Supplementary Fig. 4 | Comparison of standard microscopy vs. EpiView-D4 for brightfield imaging of standard IHC and cytology preparations of BT474, MDA-MB-453, and BT20 solid tumor xenografts. a–f, Brightfield imaging of IHC specimens stained for HER2. Specimens were prepared using standard cell block methods and IHC. Note: representative imaging regions displayed by benchtop microscope and EpiView-D4 are not the same. **g–l,** Brightfield imaging of cytology specimens. Main panels are low power fields and insets represent high power field images. Note: representative images for benchtop microscope and EpiView-D4 are of same tumor type, but from different specimens.

5) Clinical pathology of patient specimens

#	HER2 status	Cytopathology Assessment	HER2 IHC Score	HER2 Copy #	FISH Ratio
1	neg	Fibroadipose tissue; no tumor	-	-	-
6	neg	Fibroadipose tissue; no tumor	-	-	-
7	neg	Fibroadipose tissue; no tumor	-	-	-
11	neg	Fibroadipose tissue; no tumor	-	-	-
17	neg	Fibroadipose tissue; no tumor	-	-	-
18	neg	Fibroadipose tissue; no tumor	-	-	-
20	neg	Fibroadipose tissue; no tumor	-	-	-
12	neg	Tumor present	*1+*	-	-
22	neg	Tumor present	*2+*	3.2	1.6
3	neg	Tumor present	*1+*	2	1
13	neg	Tumor present	*1+*	-	-
16	neg	Tumor present	*2+*	2.8	1.2
19	neg	Tumor present	*0+*	-	-
2	pos	Tumor present	*3+*	27.2	10.2
4	pos	Tumor present	*3+*	13.4	4.9
5	pos	Tumor present	*3+*	11.1	7.9
8	pos	Tumor present	*3+*	23.9	13.5
14	pos	Tumor present	*2+*	6.3	2.2
21	pos	Tumor present	*3+*	-	-

Supplementary Table 1 | Clinical pathology of patient specimens. Tumor vs. nontumor designations were made by cytopathology assessment by a clinical pathologist. HER2 status was determined by IHC (3+ positive, 2+ equivocal, 0-1+ negative) or FISH for HER2 amplification (Positive: FISH ratio > 2.0 and HER2 gene copy # > 4.0 or HER2 gene copy # > 6.0). Values not shown for specimens identified to be non-tumor tissue by the cytopathologist.

6) Un-cropped, raw images of Western blots

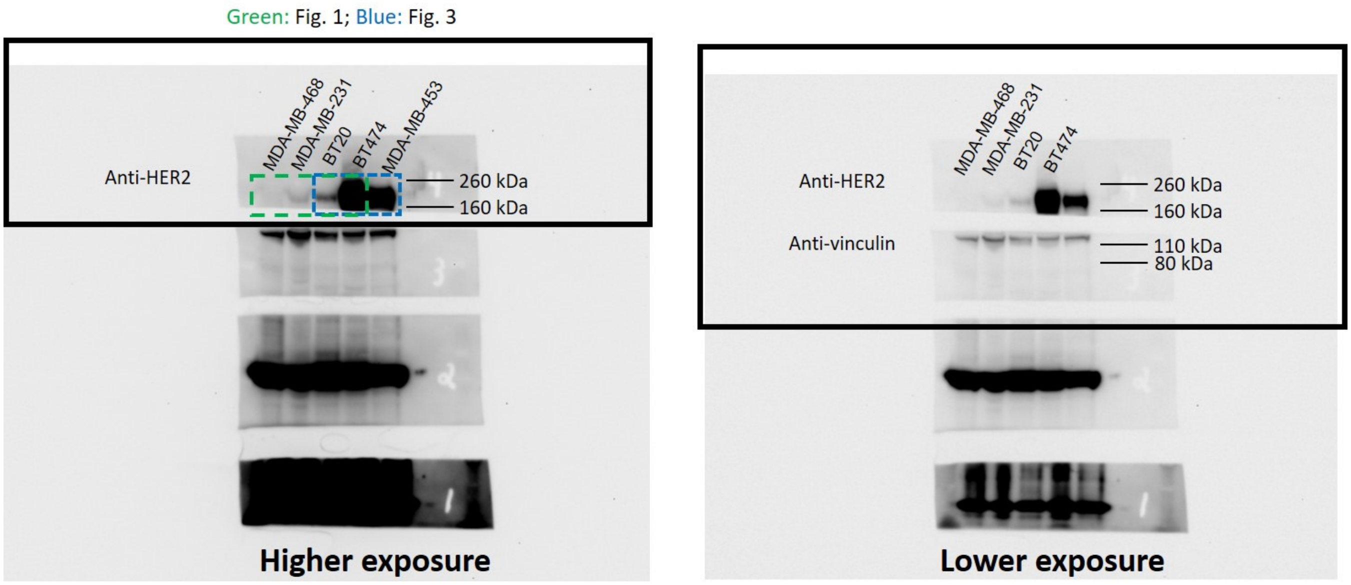

Supplementary Fig. 5 | Raw western blot images for Fig. 1 and Fig. 3 of the Main Text. Shown are the raw Western blot images used in Figures 1 & 3 of the main text, which compares HER2 expression in breast cancer cell lines. High exposure (left) and a low exposure (right) scans of the same blot are shown. Cropping used for publication is indicated by the dashed green line for Figure 1, and the dashed blue line for Figure 3. For the blot used in Figure 1, we preferred to display the expression levels of HER2 in decreasing order (not increasing order, as shown below), and therefore the left-right orientation of image is reversed and the published version. *No lane splicing or image contrast manipulation was used for any of our published data.*

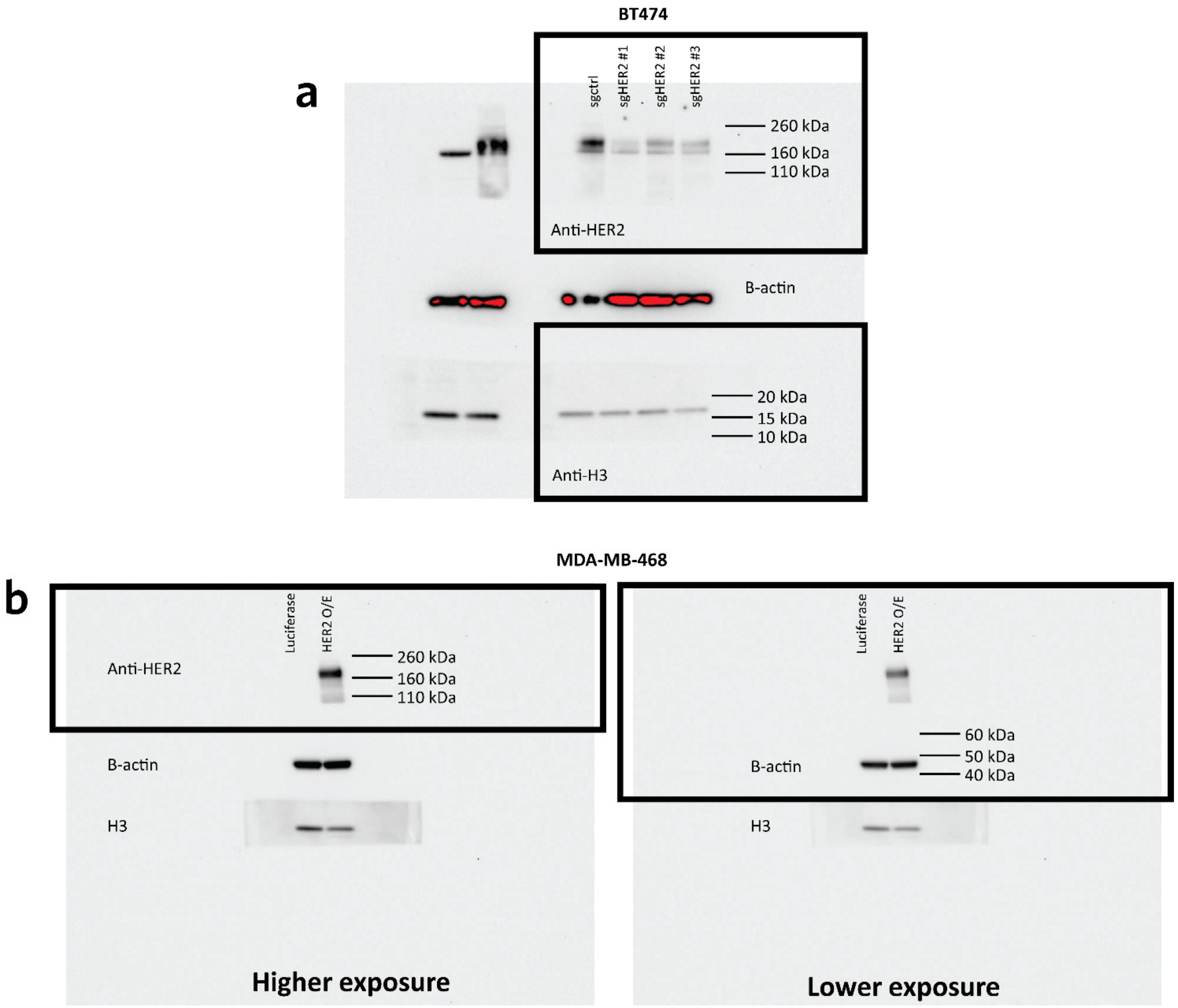

Supplementary Fig. 6 | Raw western blot images for Supplementary Fig. S1. The raw, uncropped Western blots used in Figure S1 are shown. *No lane splicing or image contrast manipulation was used for any of our published data.* **a,** Knockdown of HER2 in BT474 cells. Note that sgHER2 #3 is not included in Supplementary Fig. S1 to avoid redundancy. **b,** Overexpression of HER2 in MDA-MB-468 cells. High exposure (left) and a low exposure (right) scans of the same blot are shown.
