# Supplementary material for: Cellphone enabled point-of-care assessment of breast tumor cytology and molecular HER2 expression from fine-needle aspirates
Source: NPJ Breast Cancer. 2021 Jul 2;7:85. doi: 10.1038/s41523-021-00290-0 (PMC8253731; doi:10.1038/s41523-021-00290-0)
Supplement: Supplementary file 2 — Reporting Summary [file 41523_2021_290_MOESM2_ESM.pdf]

nature research

Corresponding author(s): Qingshan Wei, Ashutosh Chilkoti

Last updated by author(s): Mar 6, 2021

Reporting Summary

Nature Research wishes to improve the reproducibility of the work that we publish. This form provides structure for consistency and transparency in reporting. For further information on Nature Research policies, see our Editorial Policies and the Editorial Policy Checklist.

Statistics

For all statistical analyses, confirm that the following items are present in the figure legend, table legend, main text, or Methods section.

n/a	Confirmed	
☐	☒	The exact sample size (*n*) for each experimental group/condition, given as a discrete number and unit of measurement
☐	☒	A statement on whether measurements were taken from distinct samples or whether the same sample was measured repeatedly
☐	☒	The statistical test(s) used AND whether they are one- or two-sided<br>*Only common tests should be described solely by name; describe more complex techniques in the Methods section.*
☐	☒	A description of all covariates tested
☐	☒	A description of any assumptions or corrections, such as tests of normality and adjustment for multiple comparisons
☐	☒	A full description of the statistical parameters including central tendency (e.g. means) or other basic estimates (e.g. regression coefficient) AND variation (e.g. standard deviation) or associated estimates of uncertainty (e.g. confidence intervals)
☐	☒	For null hypothesis testing, the test statistic (e.g. *F*, *t*, *r*) with confidence intervals, effect sizes, degrees of freedom and *P* value noted<br>*Give P values as exact values whenever suitable.*
☒	☐	For Bayesian analysis, information on the choice of priors and Markov chain Monte Carlo settings
☒	☐	For hierarchical and complex designs, identification of the appropriate level for tests and full reporting of outcomes
☐	☒	Estimates of effect sizes (e.g. Cohen's *d*, Pearson's *r*), indicating how they were calculated

Our web collection on statistics for biologists contains articles on many of the points above.

Software and code

Policy information about availability of computer code

Data collection	Fluorescence data from D4 assays was recorded using either GenePix Pro 7 software (when using the Genepix device), or using the built-in smartphone camera app (Nokia Camera Pro) when using the EpiView-D4.
Data analysis	Images were analyzed using either updated versions of ImageJ or MATLAB software. Analytical performance of the D4 assay was done by fitting to a five-parameter logistic (5-PL) fit curve using OriginPro 9.0 (OriginLab Corp.). Statistical analysis was performed using GraphPad Prism 6.

For manuscripts utilizing custom algorithms or software that are central to the research but not yet described in published literature, software must be made available to editors and reviewers. We strongly encourage code deposition in a community repository (e.g. GitHub). See the Nature Research guidelines for submitting code & software for further information.

Data

Policy information about availability of data

All manuscripts must include a data availability statement. This statement should provide the following information, where applicable:

- Accession codes, unique identifiers, or web links for publicly available datasets
- A list of figures that have associated raw data
- A description of any restrictions on data availability

The data that support the findings of this study are either available within this Article and its Supplementary Information, or available from the corresponding author on reasonable request. Material requests should be made to chilkoti@duke.edu

Field-specific reporting

Please select the one below that is the best fit for your research. If you are not sure, read the appropriate sections before making your selection.

☒ Life sciences ☐ Behavioural & social sciences ☐ Ecological, evolutionary & environmental sciences

For a reference copy of the document with all sections, see nature.com/documents/nr-reporting-summary-flat.pdf

Life sciences study design

All studies must disclose on these points even when the disclosure is negative.

Sample size	FOR ANIMAL STUDIES: 16 specimens were collected from three different xenograft models in nude mice: BT474, MDA-MB-453, or BT20 (N = 8, 3, 5, respectively) based on sample availability. FOR HUMAN TISSUE STUDIES:ultimately 19 cases were collected and analyzed, 6 of which were confirmed to be HER2-positive, 6 HER2-negative, and 7 fibroadipose breast tissue based on specimen availability. With 3 groups comprised of 6 independent samples each, there is 81% power to detect an effect size 0f 0.75, where the effect size is the difference in means divided by the standard deviation. An effect size of 0.75 is considered moderate
Data exclusions	FOR HUMAN TISSUE STUDIES: Initially, twenty-one consecutive de-identified samples that were of sufficient size to permit FNA aspiration were selected (Duke IRB Protocol Pro00100360). Two samples had conflicting or missing pathology documentation in the medical record, and these samples were hence excluded, leading to a total of 19 specimens being analyzed for our study as described above.
Replication	The in-vitro studies were replicated on at least 5 separate days with fresh batches of assays. The animal tumor studies were replicated on at least 3 separate days with fresh batches of assays. The human tumor studies were replicated on at least 2 separate days with fresh batches of assays.
Randomization	FOR ANIMAL STUDIES: 16 specimens were collected from three different xenograft models in nude mice: BT474, MDA-MB-453, or BT20 (N = 8, 3, 5, respectively) based on sample availability. FOR HUMAN TISSUE STUDIES: The human studies were performed with previously collected tumor samples that were resected as part of routine clinical care for invasive breast cancer. Tumor samples were collected from excess tissue not required for pathologic diagnosis, then banked at -80 °C in accordance with an IRB-approved prospective tumor repository. Twenty-one consecutive de-identified samples that were of sufficient size to permit FNA aspiration were selected (Duke IRB Protocol Pro00100360). The only clinical data obtained from the tumor registry was baseline ER, PR, and HER2; two samples had conflicting or missing pathology documentation in the medical record, and these samples were hence excluded. We oversampled for HER2-positive status. Overall, 19 cases were collected and analyzed, 6 of which were confirmed to be HER2-positive, 6 HER2-negative, and 7 fibroadipose breast tissue. With 3 groups comprised of 6 independent samples each, there is 81% power to detect an effect size 0f 0.75, where the effect size is the difference in means divided by the standard deviation. An effect size of 0.75 is considered moderate. Correlation between ELISA and D4 readout was assessed using Pearson's r correlation
Blinding	The xenograft studies were not blinded. FOR HUMAN TISSUE STUDIES: HER2 status was not known to the investigator performing the EpiView-D4 analysis or the staff performing ELISA

Reporting for specific materials, systems and methods

We require information from authors about some types of materials, experimental systems and methods used in many studies. Here, indicate whether each material, system or method listed is relevant to your study. If you are not sure if a list item applies to your research, read the appropriate section before selecting a response.

Materials & experimental systems

n/a	Involved in the study	
☐	☒	Antibodies
☐	☒	Eukaryotic cell lines
☒	☐	Palaeontology and archaeology
☐	☒	Animals and other organisms
☒	☐	Human research participants
☒	☐	Clinical data
☒	☐	Dual use research of concern

Methods

n/a	Involved in the study	
☒	☐	ChIP-seq
☒	☐	Flow cytometry
☒	☐	MRI-based neuroimaging

Antibodies

Antibodies used	HER2 capture/detection Ab pairs (cat# MAB1129/AF1129, respectively, R&D Systems, Inc.)
Validation	FROM MANUFACTURER WEBSITE: Each antibody is manufactured under controlled conditions, undergoing rigorous quality control testing to ensure lot-to-lot consistency and outstanding performance in all applications listed on our datasheets. Neutralizing antibodies are tested to ensure low endotoxin levels which are reported on individual datasheets. The formulation of most antibodies does not contain azide or other preservative.All antibodies are tested for cross-reactivity with closely related molecules

using a variety of applications, including direct ELISA, to ensure specificity. These efforts are facilitated by our extensive library of in-house developed antigens.For maximum stability, most antibodies are supplied lyophilized.

Eukaryotic cell lines

Policy information about cell lines

Cell line source(s)	All established breast cancer cell lines (BT474, BT20, MDA-MB-231, MDA-MB-453, MDA-MB-468) were obtained from the Cell Culture Facility at Duke University
Authentication	Cell lines were purchased from Duke University Cell Culture Facility (CCF) and tested by the CCF for cell line identify
Mycoplasma contamination	Cell lines were tested by the Duke University Cell Culture Facility (CCF) for mycoplasma contamination
Commonly misidentified lines (See ICLAC register)	N/A

Animals and other organisms

Policy information about studies involving animals; ARRIVE guidelines recommended for reporting animal research

Laboratory animals	this study utilized female 6-week old Nu/Nu mice
Wild animals	N/A
Field-collected samples	N/A
Ethics oversight	All procedures performed were approved by the Institutional Animal Care and Use Committee at Duke University

Note that full information on the approval of the study protocol must also be provided in the manuscript.
